# Lipolysis-derived linoleic acid drives beige fat progenitor cell proliferation

**DOI:** 10.1016/j.devcel.2022.11.007

**Published:** 2022-12-05

**Authors:** Ichitaro Abe, Yasuo Oguri, Anthony R.P. Verkerke, Lauar B. Monteiro, Carly M. Knuth, Christopher Auger, Yunping Qiu, Gregory P. Westcott, Saverio Cinti, Kosaku Shinoda, Marc G. Jeschke, Shingo Kajimura

**Affiliations:** 1 Division of Endocrinology, Diabetes and Metabolism, Beth Israel Deaconess Medical Center and Harvard Medical School, Boston, MA, USA; 2 Department of Cardiology and Clinical Examination, Oita University Faculty of Medicine, Oita, Japan; 3 Laboratory of Nutrition Chemistry, Division of Food Science and Biotechnology, Graduate School of Agriculture, Kyoto University, Kyoto, Japan; 4 Faculty of Medicine, University of Toronto, Toronto, ON, Canada; 5 Biological Sciences, Sunnybrook Research Institute, Toronto, ON, Canada; 6 Department of Molecular Pharmacology, Albert Einstein College of Medicine, Bronx, NY, USA; 7 Center of Obesity, Marche Polytechnic University, Ancona, Italy; 8 Department of Medicine, Division of Endocrinology & Diabetes, Albert Einstein College of Medicine, Bronx, NY, USA; 9 Albert Einstein College of Medicine, Fleischer Institute for Diabetes and Metabolism, Bronx, NY, USA; 10 Ross Tilley Burn Centre, Sunnybrook Hospital, Toronto, ON, Canada; 11 Department of Surgery, Division of Plastic Surgery, and Department of Immunology, University of Toronto, Toronto, ON, Canada; 12 Howard Hughes Medical Institute, Chevy Chase, MD, USA; 13 Lead contact

## Abstract

*De novo* beige adipocyte biogenesis involves the proliferation of progenitor cells in white adipose tissue (WAT); however, what regulates this process remains unclear. Here, we report that in mouse models but also in human tissues, WAT lipolysis-derived linoleic acid triggers beige progenitor cell proliferation following cold acclimation, β3-adrenoceptor activation, and burn injury. A subset of adipocyte progenitors, as marked by cell surface markers PDGFRα or Sca1 and CD81, harbored cristae-rich mitochondria and actively imported linoleic acid via a fatty acid transporter CD36. Linoleic acid not only was oxidized as fuel in the mitochondria but also was utilized for the synthesis of arachidonic acid-derived signaling entities such as prostaglandin D_2_. Oral supplementation of linoleic acid was sufficient to stimulate beige progenitor cell proliferation, even under thermoneutral conditions, in a CD36-dependent manner. Together, this study provides mechanistic insights into how diverse pathophysiological stimuli, such as cold and burn injury, promote *de novo* beige fat biogenesis.

## In brief

How do diverse ‘‘browning’’ stimuli promote beige fat biogenesis? Abe et al. show that WAT lipolysis-derived linoleic acid triggers beige adipocyte progenitor cell proliferation following cold and burn injury by enhancing mitochondrial oxidation and prostanoid synthesis in progenitors.

## Graphical abstract

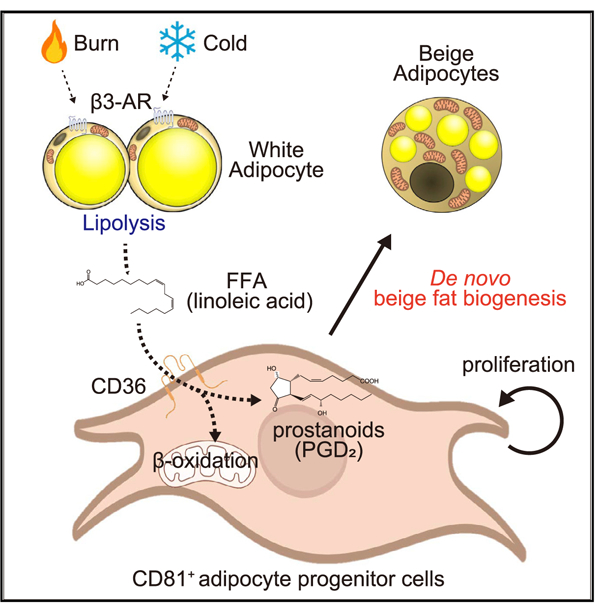

## INTRODUCTION

Adipose tissue remodeling, including lipogenesis, lipolysis, and adipogenesis, is an essential adaptative mechanism for maintaining metabolic health.^1^ The browning of white adipose tissue (WAT) is one such example: in response to chronic cold exposure and a variety of internal or external stimuli, a subset of adipocytes in the subcutaneous WAT exhibits a thermogenic phenotype, including enhanced mitochondrial biogenesis, formation of multilocular lipid droplets, and expression of uncoupling protein 1 (UCP1). The newly recruited thermogenic adipocytes, a.k.a., beige adipocytes, have caught much attention, given their potent anti-diabetic actions as seen in many animal models.^2,3^ Not only in rodents but also in adult humans, chronic activation of β3-adrenergic receptor (β3-AR) stimulates the formation of beige adipocytes in the subcutaneous WAT, concurrent with an improvement in systemic insulin sensitivity.^4,5^ Additionally, certain pathological cues, such as burn injury and cancer-associated cachexia, promote beige adipocyte biogenesis.^6–8^

Beige fat biogenesis involves *de novo* differentiation from beige adipocyte progenitor cells (APCs) and white-to-beige adipocyte conversion or reinstallation of the thermogenic program in dormant adipocytes.^9^ A recent study also reported that a subgroup of beige adipocytes can proliferate in response to β3-AR stimuli in mice.^10^ Regarding the developmental origin of *de-novo*-recruited beige fat, progenitor cells in the stromal vascular fraction (SVF) expressing *Pdgfra*, *Acta2*, *Sm22*, or *Pdgfrb* give rise to beige adipocytes.^11–16^ Human progenitor cell populations that differentiate into beige adipocytes were also reported.^17,18^ Of note, an adipogenic progenitor cell population expressing the smooth-muscle-enriched genes (*Acta2* and *Sm22*) can be isolated by using fluorescence-activated cell sorting (FACS) with a set of antibodies against cell-surface proteins, PDGFRα (human) or Sca1 (mice) and CD81.^19^ This progenitor cell population (Lin^−^: CD81^+^: PDGFRα^+^/Sca1^+^ cells: herein CD81^+^ cells) is proliferative and possesses the cell-intrinsic plasticity to differentiate to beige adipocytes *in vivo*.^19^ The identification of these markers provides new opportunities for delineating the developmental processes of *de novo* beige fat biogenesis *in vivo*.

In this work, we aim to better understand how diverse pathophysiological browning cues, such as cold acclimation, β3-AR agonist, and burn injury, promote *de novo* beige fat biogenesis. We found that WAT lipolysis-derived linoleic acid is a key downstream mediator of the browning stimuli that triggers CD81^+^ progenitor cell proliferation.

## RESULTS

### Cold and β3-AR stimuli promote CD81^+^ cell proliferation in mice and humans

**Figure 1.**
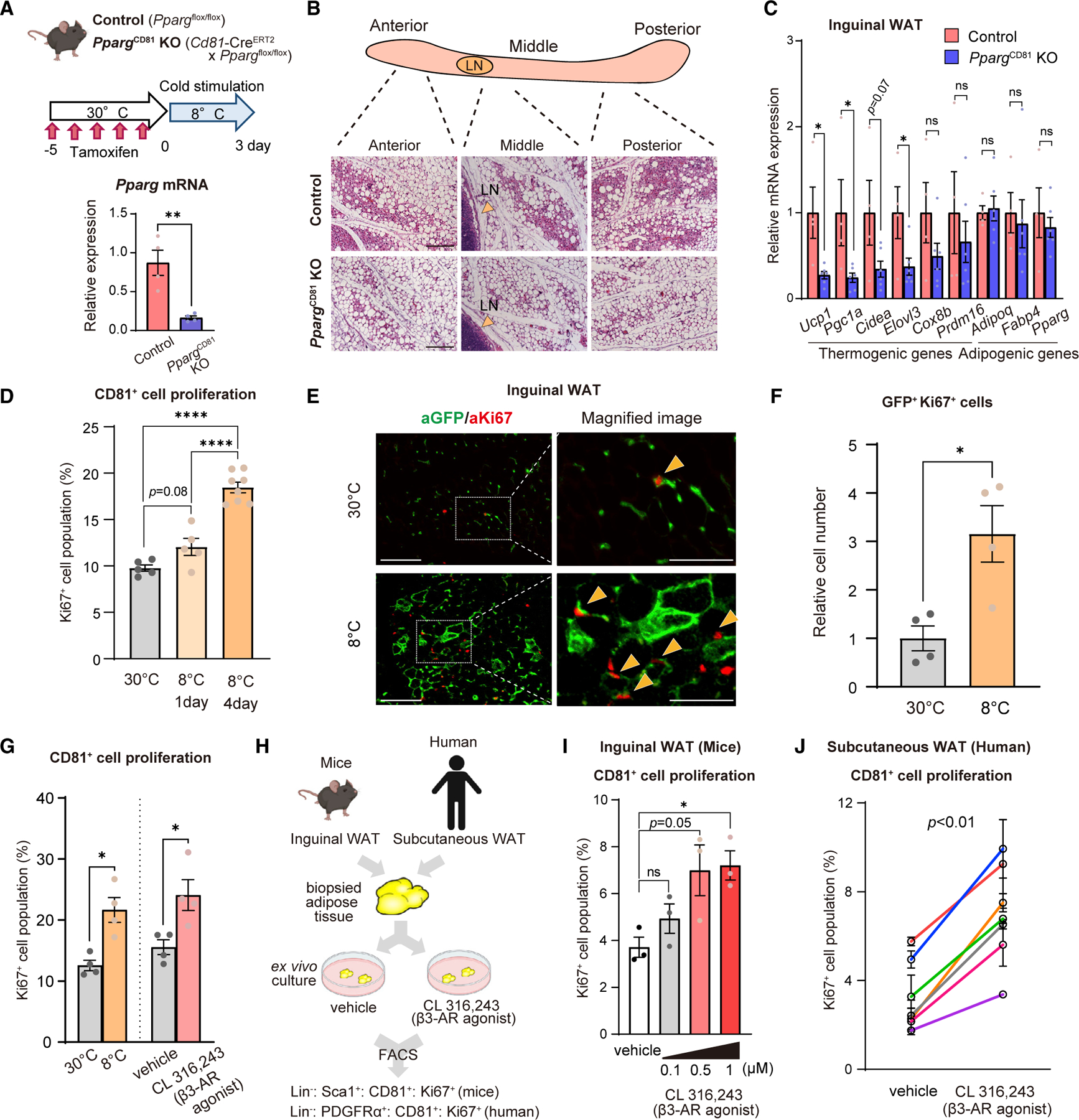
Cold and β3-AR stimuli induce CD81^+^ APC proliferation (A) Top: schematic illustration. Male mice at 10 weeks old were treated with tamoxifen at 30°C and exposed to 8°C for 3 days. Bottom: relative mRNA levels of *Pparg* in isolated CD81^+^ cells. n = 4. (B) Representative H&E staining of the inguinal WAT in (A). LN, lymph node. Scale bars, 100 μm. (C) Relative mRNA levels of indicated genes in the inguinal WAT following cold exposure. n = 6 for *Pparg*^CD81^ KO, n = 4 controls. (D) Quantification of Ki67^+^ CD81^+^ cells in the inguinal WAT of mice at 30°C and 8°C for 1 day and 4 days. n = 5 for 30°C and 8°C for 1 day, n = 8 for 8°C for 4 days. (E) Left: representative immunofluorescent staining for GFP (Green)/Ki67 (Red) in the inguinal WAT of *Cd81*-lineage reporter mice following cold exposure. Scale bars, 100 μm. Right: magnified images. The arrowheads show GFP^+^ Ki67^+^ cells. Scale bars, 50 μm. (F) Quantification of GFP^+^ Ki67^+^ cells in (E). n = 4. (G) Quantification of Ki67^+^ CD81^+^ cells in the inguinal WAT of mice. Left: mice kept at 30°C or 8°C for 3 days. Right: mice treated with vehicle or CL316,243 for 3 days. n = 4. (H) Schematic illustration of *ex vivo* studies. Mouse inguinal WAT or human subcutaneous WAT were cultured with CL316,243 (0.1–1 μM in mice, 1 μM in human sample). Ki67^+^ CD81^+^ cells in the WAT samples were quantified by FACS. (I) Quantification of mouse Ki67^+^ CD81^+^ cells in (H). n = 3. (J) Quantification of human Ki67^+^ CD81^+^ cells in (H). n = 3 technical replicates for each biopsied sample (7 pairs). p value was determined by multilevel analysis. (A, C, F, and G) *p < 0.05, **p < 0.01, by two-tailed unpaired Student’s t test. (D and I) *p < 0.05, ****p < 0.0001, by one-way ANOVA followed by the Tukey-Kramer’s post hoc test. ns, not significant.

We first determined the degree to which CD81^+^ progenitors contributed to *de novo* beige fat biogenesis by gradually acclimating mice from 30°C to 8°C for 3 days. This is based on a recent report showing that 88% of cold-induced beige adipocytes arose from *de novo* biogenesis when warm-acclimated mice at 30°C were exposed to 6°C, whereas 51% of beige adipocytes attributed to *de novo* biogenesis when mice were housed at 22°C before cold exposure.^9^ To this end, we developed mice that lacked PPARγ, the master regulator of adipogenesis, selectively in CD81^+^ cells following tamoxifen treatment (*Cd81*-Cre^ERT2^ × *Pparg*^flox/flox^), herein *Pparg*^CD81^ KO mice (Figure 1A). Note that *Cre*^*ERT2*^ was knocked into the *Cd81* gene locus, which did not affect beige adipocyte biogenesis (Figure S1A). Mice were treated with tamoxifen at 30°C and subsequently acclimated to 8°C. Following cold acclimation, control mice (*Pparg*^flox/flox^) harbored numerous clusters of UCP1-expressing multilocular adipocytes, a morphological characteristic of beige adipocytes, in the anterior, middle, and posterior regions of inguinal WAT (Figures 1B and S1B). In contrast, cold-induced beige fat biogenesis was substantially impaired in the inguinal WAT of *Pparg*^CD81^ KO mice (Figures 1B and S1B). Consistent with the morphological changes, the inguinal WAT of *Pparg*^CD81^ KO mice expressed significantly lower levels of brown/beige fat-selective genes, including *Ucp1, Pgc1α*, *Cidea*, and *Elovl3*, than that of control mice (Figure 1C). These results suggest that a large part of cold-induced *de novo* beige adipocytes originate from CD81^+^ progenitor cells in the inguinal WAT. On the other hand, we found no noticeable difference in the morphology and UCP1 expression in the interscapular BAT (iBAT) between the two groups (Figures S1C–S1F). This observation corroborates our previous study showing that CD81^+^ cells give rise to beige adipocytes in the inguinal WAT, but not to brown adipocytes in the iBAT.^19^

To determine the extent to which impaired *de novo* beige fat biogenesis influences whole-body energy homeostasis, we performed the following experiments. First, we treated *Pparg*^CD81^ KO mice and littermate controls with tamoxifen at 30°C and then transferred them to 8°C for 5 days. Subsequently, we measured their whole-body energy expenditure at 30°C in response to a single administration of CL316,243 (0.5 mg kg^−1^). Although we found no difference in VO_2_ at a basal state, *Pparg*^CD81^ KO mice exhibited modestly but significantly lower energy expenditure in response to CL316,243 relative to controls (Figure S2A). During the measurement, we did not find any difference in food intake and locomotor activity between the two groups (Figures S2B and S2C). Second, we fed *Pparg*^CD81^ KO mice and littermate controls on a high-fat diet (HFD) at room temperature for 13 weeks (Figure S2D). At the end of HFD feeding, *Pparg*^CD81^ KO mice developed impaired glucose tolerance and insulin tolerance relative to controls, although there was no difference in body weights between the genotypes (Figures S2E–S2I). This result is in agreement with previous studies in which beige fat loss leads to glucose intolerance and insulin resistance.^19–21^ We are aware of the possibility, however, that metabolic organs other than beige fat may contribute to impaired glucose homeostasis in *Pparg*^CD81^ KO mice, given the recent report that CD81 is expressed in immature pancreatic β cells,^22^ although fasting insulin levels were not different between the genotypes (Figure S2J).

Our previous study found that CD81^+^ progenitors in the SVF of inguinal WAT were proliferative in culture, and the proliferation *in vivo*, as measured by the uptake of 5-ethynyl-2-deoxyuridine (EdU), was further stimulated by cold exposure.^19^ Hence, we quantified the proliferative capacity of CD81^+^ cells using Ki67 as a proliferation marker *in vivo* (Figure S3A). We found that the number of Ki67^+^ cell population in CD81^+^ progenitors (Lin^−^: Sca1^+^: CD81^+^: Ki67^+^) in the inguinal WAT significantly increased within 3 days of cold exposure (Figures 1D and S3B). Histological analyses using *Cd81*-lineage reporter mice also showed that stromal cells in the inguinal WAT co-expressed GFP and Ki67 and that the number of Ki67^+^ GFP^+^ cells significantly increased following cold exposure (Figures 1E, 1F, and S3C). Besides cold, repeated treatment with a β3-AR agonist (CL316,243) for 3 days increased the number of Ki67^+^ CD81^+^ cells in the inguinal WAT of mice relative to vehicle-treated mice (Figure 1G).

The above result was in contrast to the observation in isolated CD81^+^ cells, wherein CL316,243 treatment in cultured CD81^+^ cells failed to stimulate the proliferation *in vitro* (Figures S3D and S3E). β1-AR signaling was previously shown to stimulate DNA synthesis, a readout of cell proliferation, in brown preadipocytes;^23^ however, norepinephrine and isoproterenol, both of which could stimulate β1-AR, did not alter CD81^+^ cell proliferation *in vitro* (Figure S3E). Accordingly, we next asked the extent to which the WAT-derived milieu, as opposed to the direct action of β3-AR signaling or circulation factors, mediates the stimulatory effect of β3-AR agonist on CD81^+^ progenitor proliferation. To this end, biopsied subcutaneous WAT depots were isolated from C57BL/6J mice (inguinal WAT) or human subjects in which CL316,243 or vehicle were added *ex vivo* (age, gender, and BMI of human subjects are listed in Table S1). We then quantified the number of Ki67^+^ CD81^+^ cells among the Lin^−^ SVFs from biopsied tissues using FACS (Figure 1H). We found that CL316,243 significantly increased the number of Ki67^+^ CD81^+^ cells in *ex vivo* cultured inguinal WAT of mice in a dose-dependent fashion (Figure 1I). In human WAT, β3-AR agonist also significantly increased the number of Ki67^+^ CD81^+^ cells (Lin^−^: PDGFRα^+^: CD81^+^: Ki67^+^) in all subjects that we examined, although the number of Ki67^+^ CD81^+^ cells at an unstimulated state varied among individuals (Figure 1J).

### Burn injury stimulates beige progenitor cell proliferation

**Figure 2.**
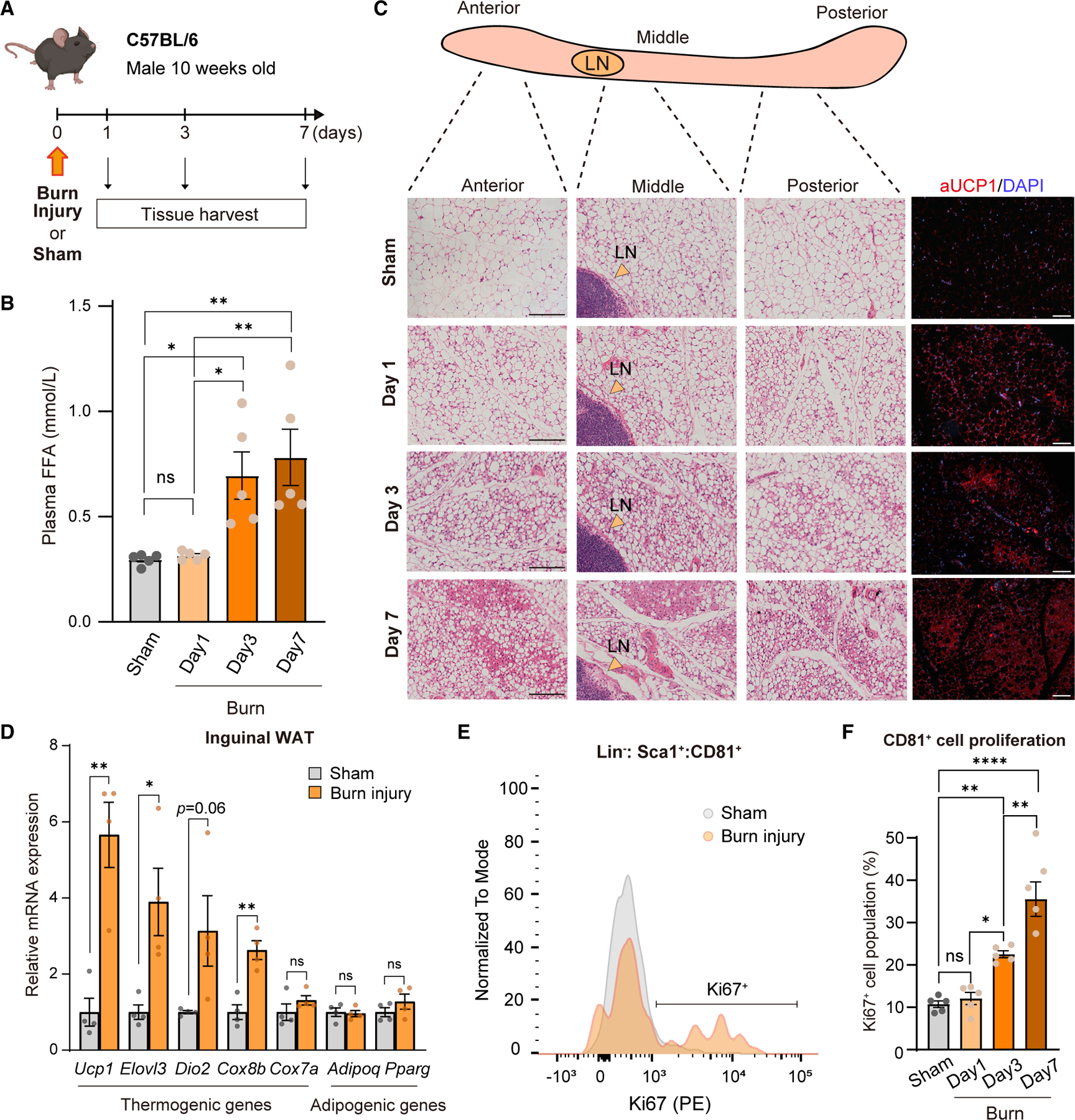
Burn injury stimulates CD81^+^ APC proliferation (A) Schematic illustration of burn injury experiments. (B) Concentrations of free fatty acids (FFAs) at indicated time points of post-burn injury. n = 5. (C) Representative H&E staining and immunofluorescent staining for UCP1 (red)/DAPI (blue) in the inguinal WAT of mice at indicated time points of post-burn injury. LN, lymph node. Scale bars, 100 μm. (D) Relative mRNA levels of indicated genes in the inguinal WAT at day 7 post-burn injury. n = 4. *p < 0.05, **p < 0.01, by two-tailed unpaired Student’s t test. (E) Representative FACS histogram images of Ki67^+^ CD81^+^ cell population in the inguinal WAT of sham and burn injury at day 7 post-burn injury. (F) Quantification of Ki67^+^ CD81^+^ cells in the inguinal WAT of mice. n = 5. (B and F) *p < 0.05, **p < 0.01, ****p < 0.0001, by one-way ANOVA followed by the Tukey-Kramer’s post hoc test.

Burn injury is a powerful pathological stimulus of beige fat biogenesis and a hypermetabolic state;^6,24^ however, the developmental process remains poorly characterized. Following the established protocol in mice,^25^ we harvested inguinal WAT depots of C57BL/6J mice at 24 h (1 day), 3 days, and 7 days after burn injury or sham (Figure 2A). Plasma free fatty acid (FFA) levels were significantly elevated at 3 and 7 days after burn injury compared with sham control mice (Figure 2B). At 7 days post-burn injury, the inguinal WAT of injured mice harbored copious clusters of beige adipocytes with multilocular adipocytes, many of which expressed UCP1 protein, in all the regions (Figure 2C). The morphological change was accompanied by increased expression of brown/beige fat-selective genes, such as *Ucp1*, *Elovl3*, *Dio2*, and *Cox8b* at 7 days of post-burn injury relative to sham control mice (Figure 2D). Importantly, we found that the number of Ki67^+^ CD81^+^ cells in the inguinal WAT significantly increased at 3 and 7 days of post-burn injury (Figures 2E and 2F). These results suggest that a pathological stimulus of beige fat biogenesis involves the proliferation of CD81^+^ progenitor cells in the inguinal WAT.

### Lipolysis is required for beige fat progenitor cell proliferation

**Figure 3.**
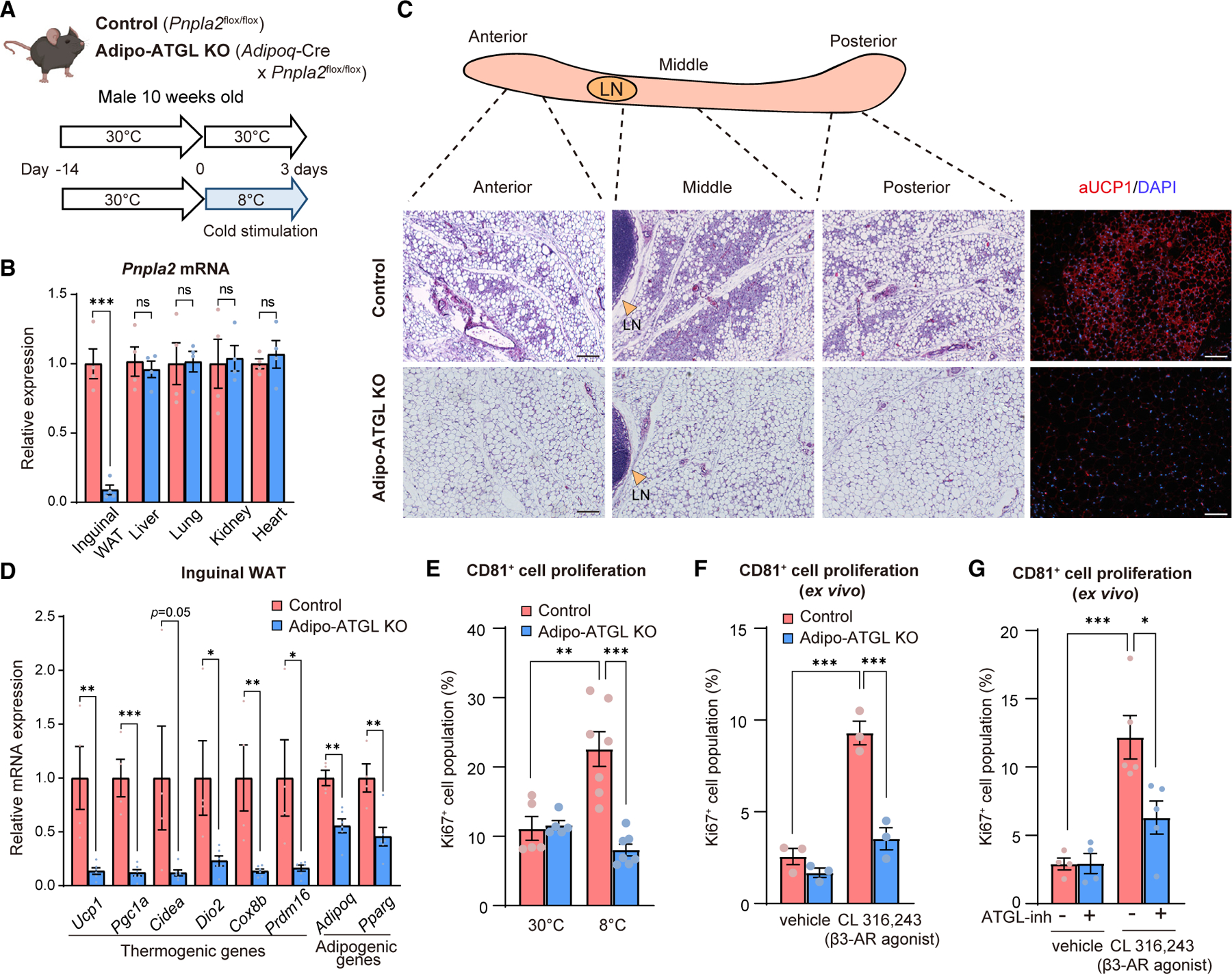
Lipolysis is required for CD81^+^ APC proliferation (A) Schematic illustration of the experiments. (B) Relative mRNA levels of *Pnpla2* in indicated tissues. n = 4. (C) Representative H&E staining and immunofluorescent staining for UCP1 (red)/DAPI (blue) in the inguinal WAT of mice. LN, lymph node. Scale bars, 100 μm. (D) Relative mRNA levels of indicated genes in the inguinal WAT. n = 6 for Adipo-ATGL KO mice, n = 4 for controls. (E) Quantification of Ki67^+^ CD81^+^ cells in the inguinal WAT. n = 5 at 30°C, n=7 at 8°C. (F) Quantification of Ki67^+^ CD81^+^ cells in *ex vivo* cultured inguinal WAT. Adipose tissues were treated with vehicle or CL316,243 at 1 μM. n = 3. (G) Quantification of Ki67^+^ CD81^+^ cells in *ex vivo* cultured inguinal WAT that were treated with atglistatin at 50 μM and/or CL316,243 at 1 μM. n = 4 for vehicle, and n = 5 for CL316,243. (B and D) *p < 0.05, **p < 0.01, ***p < 0.001, by two-tailed unpaired Student’s t test. (E–G) *p < 0.05, **p < 0.01, ***p < 0.001, by one-way ANOVA followed by the Tukey-Kramer’s post hoc test.

Both cold exposure and burn injury stimulate adipose tissue lipolysis and the release of FFAs into the circulation.^26,27^ Based on our results, wherein the β3-AR signal activated beige progenitor cell proliferation in *ex vivo* WAT but not in isolated CD81^+^ cells, we hypothesized that WAT lipolysis-derived factors mediated beige progenitor cell proliferation. To test the hypothesis, we used mice that lacked adipose triglyceride lipase (ATGL), a crucial enzyme that catalyzes the initial step of intracellular triglyceride hydrolysis,^28^ in an adipocyte-specific manner (adiponectin-Cre × *Pnpla2*^flox/flox^), henceforth Adipo-ATGL KO mice. In this experiment, Adipo-ATGL KO mice and littermate controls (*Pnpla2*^flox/flox^) were housed at 30°C for 14 days and subsequently exposed to 8°C for 3 days (Figures 3A and 3B). Mice had full access to food and water during cold exposure as Adipo-ATGL KO mice would develop hypothermia under a fasted condition.^29^

Following 3-day-cold acclimation, histological analysis of the inguinal WAT depots from control mice found numerous beige adipocytes with multilocular lipids and UCP1 expression, particularly in the middle region. In contrast, cold-induced beige adipocyte biogenesis was near completely blunted in Adipo-ATGL KO mice (Figure 3C). Furthermore, adipocyte-specific ATGL loss significantly reduced the expression of the brown/beige fat-selective genes, including *Ucp1*, *Pgc1a*, *Cidea*, *Dio2*, *Cox8b*, and *Prdm16*, in the inguinal WAT (Figure 3D). In agreement with the previous study,^29^ the iBAT of Adipo-ATGL KO mice contained much larger lipid droplets and expressed lower levels of UCP1 protein than those in control mice (Figures S4A–S4D).

Next, we used FACS to examine CD81^+^ cell proliferation in the inguinal WAT of mice kept at 30°C and after cold acclimation. At 30°C, there was no difference in the number of Ki67^+^ CD81^+^ cells between the genotypes. Cold acclimation significantly increased the number of Ki67^+^ CD81^+^ cells in control mice; however, this cold-induced beige progenitor cell proliferation was completely blunted in Adipo-ATGL KO mice (Figures 3E and S4E). When *ex vivo* cultured inguinal WAT were treated with CL316,243, we also observed a significant increase in the number of Ki67^+^ CD81^+^ cells in control mice-derived WAT depots. In contrast, the stimulatory effect of CL316,243 was blunted in Adipo-ATGL KO mice (Figure 3F). In compatible with the genetic studies, pharmacological inhibition of ATGL by Atglistatin potently blocked the stimulatory effect of CL316,243 on CD81^+^ cell proliferation in the inguinal WAT of wild-type mice (Figure 3G).

### Linoleic acid stimulates beige fat progenitor cell proliferation

**Figure 4.**
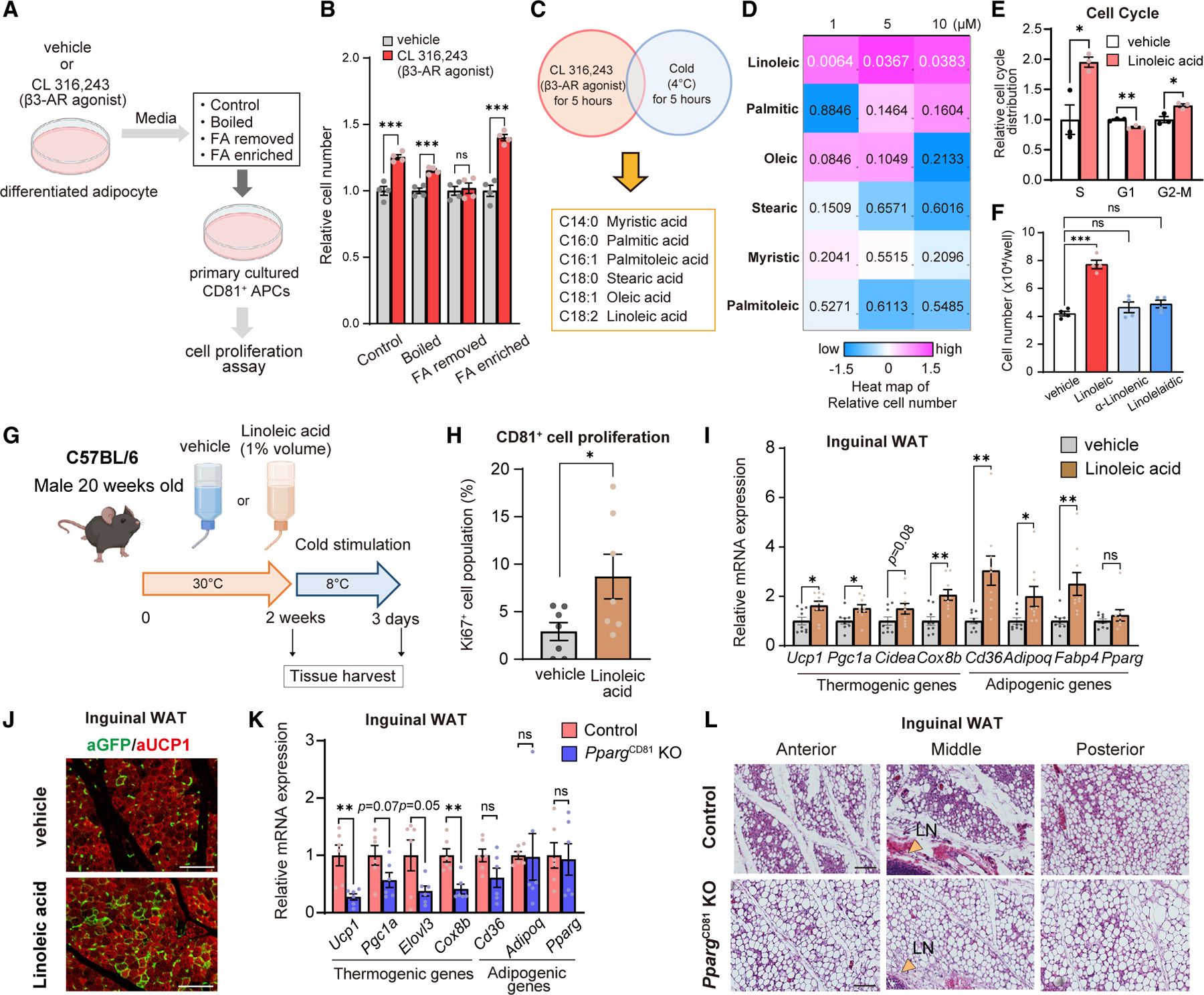
Linoleic acid stimulates CD81^+^ APC proliferation (A) Schematic illustration of the experiments. See text for details. (B) Relative cell number (normalized to each vehicle) of inguinal WAT-derived CD81^+^ cells in (A). n = 4. (C) Candidates of fatty acids studied in the study. (D) Heatmap of cell numbers in CD81^+^ cells treated with indicated fatty acids for 4 days. The color scale shows *Z* score levels of the relative cell number. n = 4. p value by two-way repeated-measures ANOVA is shown in each panel. (E) Quantification of CD81^+^ cells in indicated cell cycle phases. n = 3. (F) The cell number of inguinal WAT-derived CD81^+^ cells treated with indicated molecules for 4 days. n = 4. ***p < 0.001 by one-way ANOVA followed by the Tukey-Kramer’s post hoc test. (G) Schematic illustration of linoleic acid supplementation. (H) Quantification of Ki67^+^ CD81^+^ cells in the inguinal WAT following linoleic acid supplementation at 30°C. n = 7. (I) Relative mRNA levels of indicated genes in the inguinal WAT following cold exposure. n = 9. (J) Representative immunofluorescent staining for GFP (green)/UCP1 (red) in the inguinal WAT of *Cd81*-lineage reporter mice following linoleic acid supplementation and cold exposure. Scale bars, 100 μm. (K) Relative mRNA levels of indicated genes in the inguinal WAT following linoleic acid supplementation and cold exposure. n = 6. (L) Representative H&E staining in the inguinal WAT. LN, lymph node. Scale bars, 100 μm. (B,E, H, I, and K) *p < 0.05, **p < 0.01, ***p < 0.001, by two-tailed unpaired Student’s t test. ns, not significant.

We devised the following strategy to identify lipolysis-derived factors that mediated beige progenitor cell proliferation (Figure 4A). First, we collected serum-free culture media from differentiated adipocytes that were treated with CL316,243 or vehicle. A part of the media was filtered and then added to primary CD81^+^ cells isolated from the inguinal WAT of wild-type mice. The remaining media was either heated at 95°C for 10 min (boiled) or treated with chloroform/methanol to remove or extract fatty acids. Second, we tested how these media affected CD81^+^ cell proliferation in culture. We found that boiled media retained the ability to stimulate CD81^+^ cell proliferation. On the other hand, removing lipids by chloroform/methanol abolished the proliferative activity of the culture media. Notably, lipid extracts from the media were able to stimulate CD81^+^ cell proliferation (Figure 4B).

Accordingly, we searched for published lipidomics datasets in adipose tissue and circulating lipid profiles in response to cold exposure and β3-AR stimulation.^26,30,31^ Special attention was paid to 6 fatty acid species whose levels consistently increased both by cold exposure and β3-AR agonist (Figure 4C). Then, we treated inguinal WAT-derived primary CD81^+^ cells with BSA-conjugated fatty acid at concentrations of 1, 5, and 10 μM. We found that supplementation of linoleic acid (18:2 n–6) at all doses significantly stimulated CD81^+^ cell proliferation, whereas the effects of other fatty acids did not reach statistical significance (Figure 4D). Subsequently, we validated the effect of linoleic acid in independent assays with several FBS concentrations (Figure S5A). Linoleic acid treatment promoted cell proliferation by increasing the number of cells in the S and G2-M phases, preferentially in CD81^+^ cells (Figures 4E, S5B, and S5C). We also found that linoleic acid, but not α-linolenic acid (18:3 n–3) or linolelaidic acid, a geometric isomer of linoleic acid, stimulated CD81^+^ cell proliferation (Figures 4F and S5D).

Linoleic acid is an essential fatty acid in rodents and humans, such that it derives primarily from dietary sources. Hence, we examined if oral supplementation of linoleic acid promoted beige progenitor cell proliferation *in vivo*. To test this, wild-type male mice (20 weeks old) at 30°C were orally supplemented with linoleic acid (1% volume) or vehicle for 2 weeks (Figure 4G). LC-MS analyses confirmed that the oral supplementation led to elevated linoleic acid levels in the inguinal WAT (Figure S6A). We then harvested the inguinal WAT of these mice and quantified the number of proliferative Ki67^+^ CD81^+^ cells by FACS-based assays. Oral linoleic acid supplementation significantly increased the number of Ki67^+^ CD81^+^ cells in the inguinal WAT by nearly 3-fold in mice kept at 30°C (Figure 4H). Similarly, 2-week supplementation of linoleic acid in 8-month-old mice (32 weeks old) at 30°C significantly stimulated CD81^+^ cell proliferation in the inguinal WAT (Figure S6B). Of note, linoleic acid supplementation alone did not promote beige adipocyte differentiation at 30°C, although it sufficiently increased progenitor cell proliferation (Figure S6C). This result suggests that lipolysis-derived linoleic acid can trigger progenitor cell proliferation without cold stimuli, but it is not a sufficient stimulus for beige adipocyte differentiation. Thus, we next asked if an increased progenitor pool led to enhanced the WAT browning capacity. To this end, mice supplemented with linoleic acid or vehicle at 30°C were gradually acclimated to 8°C for 3 days. Although cold acclimation to 8°C stimulated beige fat biogenesis of vehicle-treated mice, linoleic acid supplementation further enhanced beige fat biogenesis in the inguinal WAT (Figures 4I and S6D).

We next performed the following complementary experiments to determine the degree to which CD81^+^ cells contributed to beige fat biogenesis in response to linoleic acid supplementation. First, we supplemented *Cd81*-lineage reporter mice with linoleic acid or vehicle at 30°C for 2 weeks and subsequently transferred them to 8°C (Figure S6E). We found that linoleic acid supplementation enhanced CD81^+^ cell-derived beige adipocytes in the inguinal WAT (Figure 4J). Second, we supplemented control and *Pparg*^CD81^ KO mice with linoleic acid (Figure S6F). We found that linoleic acid supplementation potently stimulated beige fat biogenesis in the inguinal WAT of control mice, whereas the stimulatory effect was significantly blunted in *Pparg*^CD81^ KO mice (Figure 4K). In alignment with the molecular analysis, histological analyses of the inguinal WAT showed that *Pparg*^CD81^ KO mice harbored fewer multilocular beige adipocytes than control mice (Figure 4L). These results suggest that linoleic acid supplementation potentiates CD81^+^ cell-derived *de novo* beige fat biogenesis when combined with cold acclimation.

### Active mitochondrial metabolism and arachidonic acid pathway in beige fat progenitors

**Figure 5.**
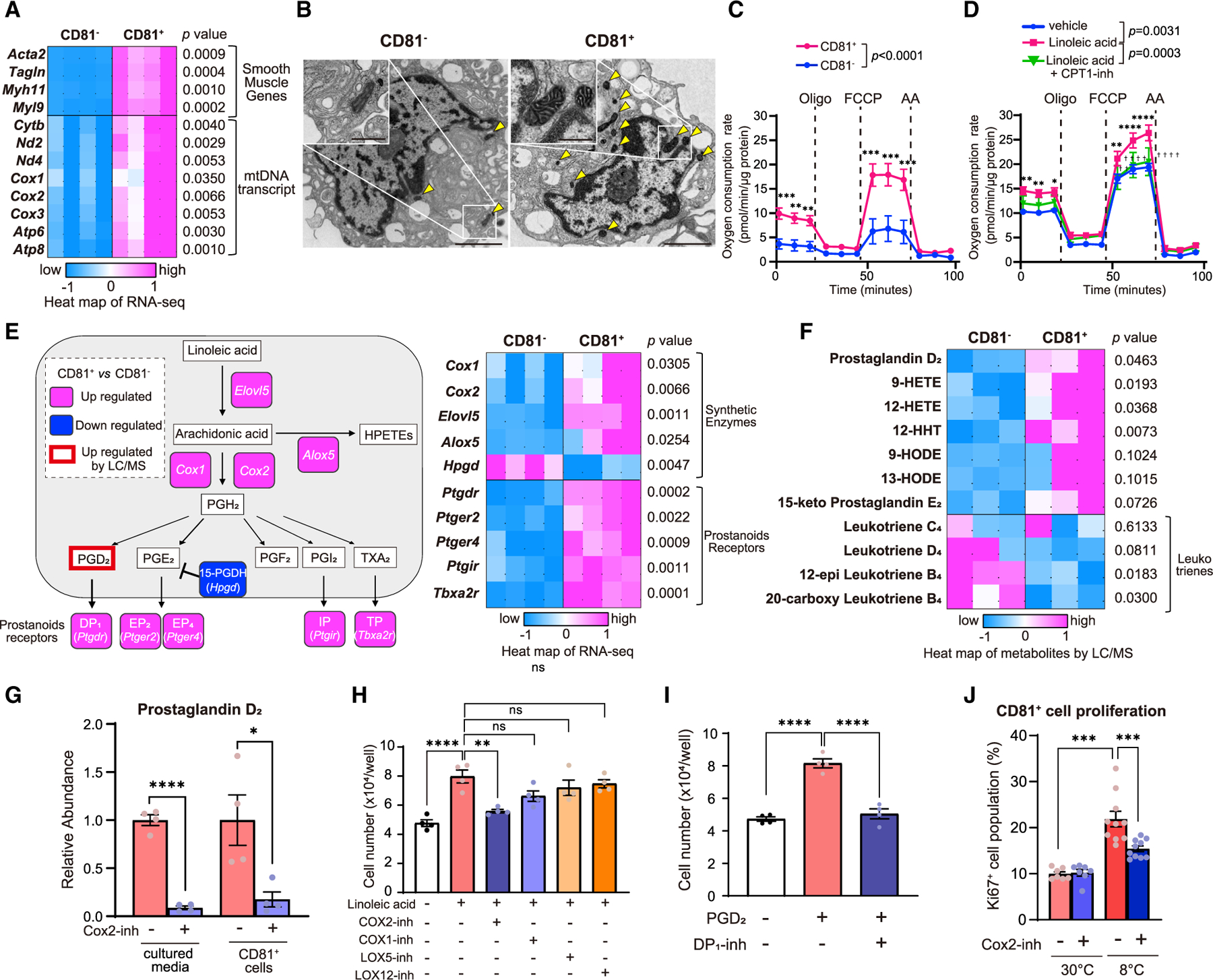
CD81^+^ APC is enriched in mitochondrial metabolism and arachidonic acid pathway (A) Heatmap of transcriptome in CD81^+^ cells and CD81^−^ cells from the inguinal WAT. The color scale shows *Z* score levels of each gene. n = 4. (B) Representative transmission electron microscopy images of CD81^+^ cells and CD81^−^ cells in the inguinal WAT. Yellow arrowheads indicate the mitochondria. Scale bars, 2 μm. Inset: magnified images of the mitochondria. Scale bars, 0.5 μm. (C) Changes in OCR in inguinal WAT-derived CD81^+^ cells and CD81^−^ cells treated with indicated compounds. n = 8 for CD81^+^ cells, n = 10 for CD81^−^ cells. **p < 0.01, ***p < 0.001, by two-way repeated-measures ANOVA followed by two-tailed unpaired Student’s t test. (D) Changes in OCR in inguinal WAT-derived CD81^+^ cells treated with indicated molecules. n = 5. *p < 0.05, **p < 0.01, ****p < 0.0001 (versus vehicle), and ^☨☨^p < 0.01, ^☨☨☨☨^p < 0.0001 (versus linoleic acid), by two-way repeated-measures ANOVA followed by two-tailed unpaired Student’s t test. (E) Left: upregulated (red) and downregulated (blue) genes in CD81^+^ cells relative to CD81^−^ cells. Right: heatmap of indicated genes as shown by *Z* score levels. n = 4. (F) Heatmap of indicated arachidonic acid-derived lipids in CD81^+^ cells and CD81^−^ cells isolated from mice following cold exposure. n = 3. (G) Relative levels of PGD_2_ in primary CD81^+^ cells and released PGD_2_ into the media. Cells were incubated with linoleic acid with or without a Cox2 inhibitor NS-398. n = 4. (H and I) The number of inguinal WAT-derived CD81^+^ cells treated with indicated compounds. n = 4. (J) Quantification of Ki67^+^ CD81^+^ cells in the inguinal WAT of mice treated with vehicle or celecoxib. n = 7 for 30°C, n = 10 at 8°C. (A and E–G) *p < 0.05, ****p < 0.0001, by two-tailed unpaired Student’s t test. (H–J) **p < 0.01, ***p < 0.001, ****p < 0.0001, by one-way ANOVA followed by the Tukey-Kramer’s post hoc test.

To uncover the mechanism by which linoleic acid stimulated beige progenitor cell proliferation, we performed RNA-seq of primary isolated CD81^+^ cells (Lin^−^: Sca1^+^: CD81^+^) and CD81^−^ cells (Lin^−^: Sca1^+^: CD81^−^) in the inguinal WAT. In agreement with our previous work,^19^ the transcriptomics analysis showed that CD81^+^ cells expressed higher levels of smooth-muscle enriched genes, including *Acta2*, *Sm22*, and *Myh11*, compared with CD81^−^ cells (Figure 5A). The subsequent analysis validated the previous work, wherein pathways related to the focal adhesion kinase (FAK) signaling, ECM-receptor interaction, and vascular smooth muscle were upregulated in CD81^+^ cells relative to CD81^−^ cells (Figure S6G). Notably, CD81^+^ cells expressed significantly higher levels of mitochondria-encoded genes, such as *Atp6*, *Atp8*, *Cox1*, *Cox2*, and *Cytb*, than CD81^−^ cells derived from the inguinal WAT (Figure 5A). Electron microscopy (EM) analyses found that CD81^+^ cells harbored numerous mitochondria, many of which were spherical or elliptical in shape and parallel orientation of dense cristae, showing the morphological characteristics of brown preadipocytes^32^ (Figure 5B). In contrast, mitochondria in CD81^−^ cells were generally less dense in the cristae and showed a white fat-like morphology. Moreover, the oxygen consumption rate of CD81^+^ cells was significantly higher than that of CD81^−^ cells at the basal state and following carbonyl cyanide 4-(trifluoromethoxy) phenylhydrazone (FCCP) treatment (Figure 5C). Linoleic acid supplementation further activated the bioenergetics of CD81^+^ cells through enhanced FA oxidation, as inhibition of FA oxidation by a carnitine palmitoyltransferase (CPT) inhibitor (etomoxir) blunted the stimulatory effect of linoleic acid on cellular respiration (Figure 5D).

The above result suggests that CD81^+^ beige progenitor cells actively utilized linoleic acid for mitochondrial β-oxidation. However, it does not fully explain the specificity of action, i.e., why did linoleic acid, but not other fatty acids, stimulate CD81^+^ cell proliferation? A hint arose from the transcriptome data in which the arachidonic acid synthesis pathway was highly enriched in CD81^+^ cells compared with CD81^−^ cells (Figure 5E). For instance, the expressions of *Elvol5*, *Cox1*, and *Cox2*, the essential enzymes for the synthesis of prostanoids, were significantly higher in CD81^+^ cells than CD81^−^ cells. In addition, CD81^+^ cells expressed higher levels of prostanoid receptors that were known to stimulate cAMP signaling, including prostaglandin D_2_ (PGD_2_) receptor (DP1, encoded by *Ptgdr1*), prostaglandin E_2_ (PGE_2_) receptors (EP2, encoded by *Ptger2*, and EP4, encoded by *Ptger4*), prostacyclin receptor (IP, encoded by *Ptgir*), and prostanoid TP receptor (TXA2-R, encoded by *Tbxa2r*). In contrast, CD81^+^ expressed lower levels of 15-hydroxyprostaglandin dehydrogenase (15-PGDH), the PGE_2_-degrading enzyme, than CD81^−^ cells. Thus, we next employed lipidomics and examined the levels of prostaglandins in primary CD81^+^ and CD81^−^ cells isolated from mice that were exposed to 8°C for 3 days. The analysis found significantly higher levels of PGD_2_ in CD81^+^ cells relative to CD81^−^ cells (Figure 5F). Besides, CD81^+^ cells contained higher levels of several arachidonic-acid-derived metabolites, including 9- and 12-hydroxyeicosatetraenoic acid (9-HETE and 12-HETE) and 12-hydroxyheptadecatrenoic acid (12-HHT) relative to CD81^−^ cells. There was also a trend of increase in 9- and 13-hydroxyoctadecadienoic acid (9-HODE and 13-HODE) and 15-keto PGE_2_ versus CD81^−^ cells, although the difference was not statistically significant. On the other hand, leukotriene B_4_ (12-epi leukotr ine B_4_ and 20-carboxy leukotriene B_4_), which are eicosanoid inflammatory mediators through the action of arachidonate 5-lipoxygenase (Alox5), were lower in CD81^+^ cells than in CD81^−^ cells.

The elevated prostaglandin synthetic pathway was intriguing as previous studies demonstrated that prostanoids via the Cox2 pathway mediated cold-induced beige fat biogenesis in mice.^33–36^ Accordingly, we next incubated primary CD81^+^ cells with linoleic acid in a serum-free medium together with or without a Cox2 inhibitor, NS-398. Subsequently, we employed liquid chromatography-tandem mass spectrometry (LC-MS/MS) and quantified PGD_2_ contents in CD81^+^ cells and the released PGD_2_ levels into the media. We found that Cox2 inhibition significantly reduced both the cellular and released levels of PGD_2_ (Figure 5G), suggesting that Cox2 is required for the synthesis of PGD_2_ in CD81^+^ cells.

To examine how linoleic acid stimulates CD81^+^ beige progenitor cell proliferation, we performed the following experiment. First, we treated primary isolated CD81^+^ cells with linoleic acid in the presence or absence of selective inhibitors for Cox1 (SC-560) or Cox2 (NS-398) to block the prostanoid synthesis. Additionally, we co-treated CD81^+^ cells with selective inhibitors for Alox5 (MK-886) and Alox12 (Baicalein) to block the synthesis of HETEs. We found that linoleic acid stimulated CD81^+^ cell proliferation, whereas the Cox2 inhibitor significantly blunted the stimulatory effect of linoleic acid. On the other hand, inhibitors for Cox1, Alox5, and Alox12 did not interfere with the effect of linoleic acid (Figure 5H). Second, we determined the extent to which PGD_2_ directly acted on beige progenitor cells, given the data that CD81^+^ cells expressed high levels of prostanoid receptors, such as DP1. We found that PGD_2_ potently stimulated CD81^+^ cell proliferation, whereas a specific inhibitor for DP1 (MK-0524) abrogated the stimulatory effect of PGD_2_ (Figure 5I). Third, we asked if the Cox2-mediated prostanoid synthesis pathway was required for beige progenitor cell proliferation *in vivo*. To this end, we treated mice at 30°C with Cox2 inhibitor (celecoxib at 15 mg kg^−1^ day^−1^) or vehicle for 8 days, and subsequently, a subset of these mice was exposed to 8°C for 3 days. We found that cold exposure potently increased the number of Ki67^+^ CD81^+^ cells in the inguinal WAT; however, Cox2 inhibitor treatment significantly blunted the stimulatory effect of cold exposure on CD81^+^ progenitor cell proliferation (Figure 5J). These results suggest that lipolysis-derived linoleic acid is oxidized by CD81^+^ progenitor cells in the mitochondria and also used for the synthesis of PGD_2_ via the Cox2 pathway.

### CD36 is required for beige progenitor cell proliferation following cold and linoleic acid

**Figure 6.**
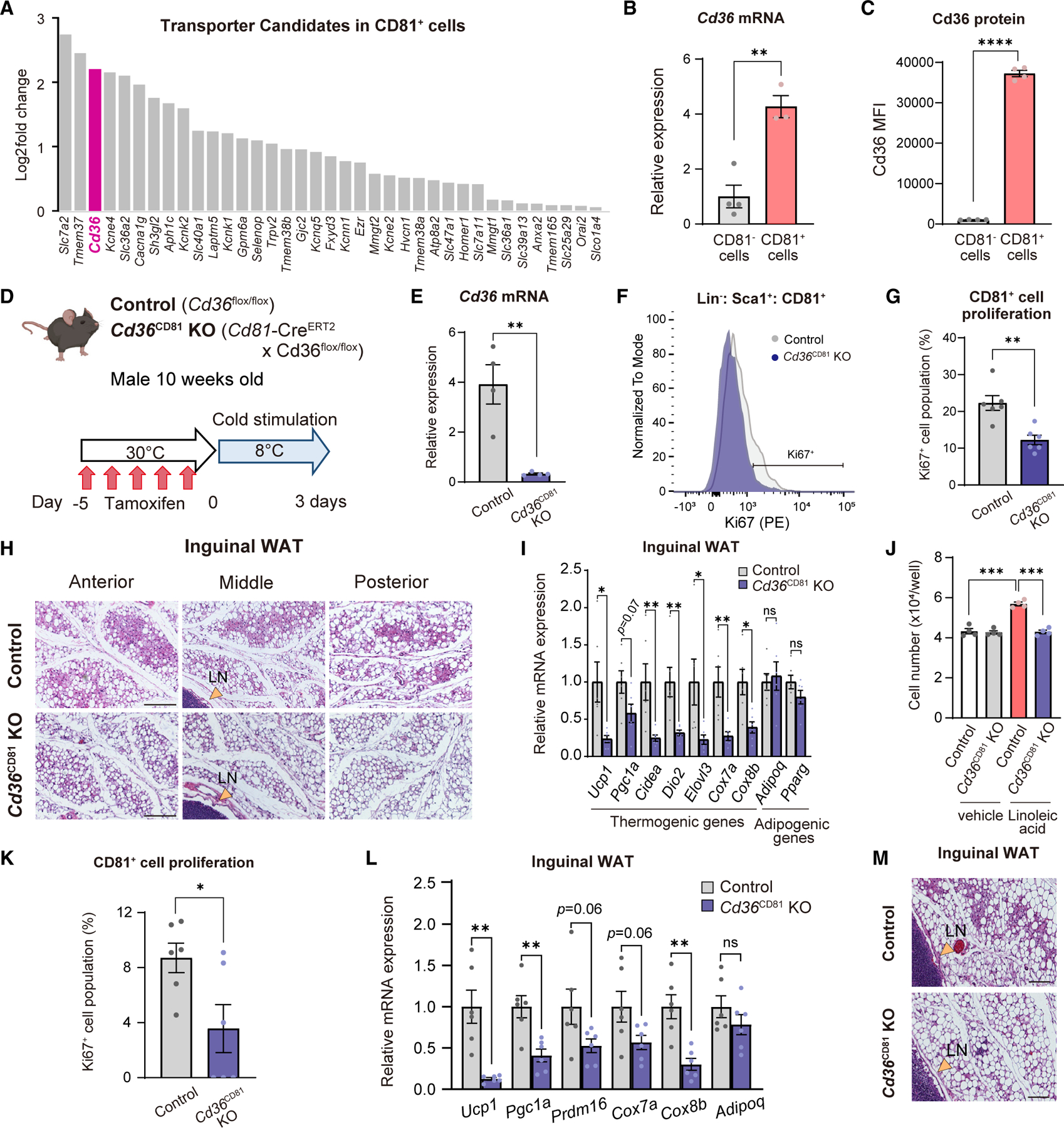
CD36 is required for CD81^+^ APC proliferation (A) Relative expression levels of indicated genes enriched in inguinal WAT-derived CD81^+^ cells relative to CD81^−^ cells. (B) Relative mRNA levels of *Cd36* in CD81^+^ cells and CD81^−^ cells. n = 4. (C) Relative Cd36 protein expression in CD81^+^ cells and CD81^−^ cells. n = 4. (D) Schematic illustration of the experiments. (E) Relative mRNA levels of *Cd36* in the inguinal WAT-derived CD81^+^ cells. n = 4. (F) Representative FACS histogram of Ki67^+^ CD81^+^ cells in (D). (G) Quantification of Ki67^+^ CD81^+^ cells in (F). n = 6. (H) Representative H&E staining in the inguinal WAT of mice in (D). LN, lymph node. Scale bars, 100 μm. (I) Relative mRNA levels of indicated genes in the inguinal WAT. n = 6. (J) The number of primary CD81^+^ cells treated with vehicle or linoleic acid for 4 days. n = 4. ***p < 0.001 by one-way ANOVA followed by the Tukey-Kramer’s post hoc test. (K) Quantitative analysis of Ki67^+^ CD81^+^ cells in the inguinal WAT following linoleic acid supplementation at 30°C. n = 6. (L) Relative mRNA levels of indicated genes in the inguinal WAT following linoleic acid supplementation and cold exposure. n = 6. (M) Representative H&E staining in the inguinal WAT. Scale bars, 100 μm. (B, C, E, G, I, K, and L) *p < 0.05, **p < 0.01, ****p < 0.0001, by two-tailed unpaired Student’s t test. ns, not significant.

Next, we aim to address how beige progenitor cells uptake linoleic acid. First, we searched for cell-membrane proteins that were highly expressed in CD81^+^ beige progenitor cells relative to CD81^−^ cells in the inguinal WAT. The transcriptomics data identified several candidates, including *Slc7a2* (or known as Cat2), *Tmem37*, and *Cd36* (Figure 6A). CD36 caught our attention, given the known role of fatty acid uptake.^37,38^ It is also notable that CD36 forms a heteromeric complex with CD81, integrin β1 or β2, and Src-family kinases that drive the internalization of CD36.^39^ Thus, we validated this result in independent samples by using qPCR (Figure 6B) and its protein expression by using a CD36 antibody (Figure 6C).

Next, we tested the hypothesis that CD36 mediated the uptake of linoleic acid in beige progenitor cells by deleting *Cd36* selectively in CD81^+^ cells (*Cd81*-Cre^ERT2^ × *Cd36*^flox/flox^ mice, herein *Cd36*^CD81^ KO mice). *Cd36*^CD81^ KO mice and littermate controls were treated with tamoxifen at 30°C and then gradually acclimated to 8°C for 3 days (Figures 6D and 6E). Following 3-day-cold exposure, we found that the number of Ki67^+^ CD81^+^ cells in the inguinal WAT of *Cd36*^CD81^ KO mice was significantly lower than that of control mice by 45% (Figures 6F and 6G). Histological analyses showed that the inguinal WAT of *Cd36*^CD81^ KO mice harbored fewer multilocular UCP1^+^ beige adipocytes than that of control mice (Figures 6H and S7A). It is worth pointing out, however, that the difference between *Cd36*^CD81^ KO mice and controls was less profound than what we observed in Adipo-ATGL KO mice, suggesting that CD36 was required for CD81^+^ beige progenitor cell proliferation, whereas CD36 loss in CD81^+^ cells did not impact beige adipocyte biogenesis from other lineages or reinstallation of the thermogenic program in dormant adipocytes.^9,10,12,40^ Nonetheless, impaired beige fat biogenesis by CD36 loss was significant, given the lower expression levels of the brown/beige-fat selective genes in the inguinal WAT of *Cd36*^CD81^ KO mice relative to those of controls (Figure 6I). In contrast, we did not find a noticeable difference in the morphology and the mRNA levels of brown fat-selective genes in the BAT (Figures S7B and S7C).

Finally, we performed the following studies to determine whether linoleic-acid-induced beige progenitor cell proliferation depended on CD36. First, we examined cell proliferation of primary CD81^+^ cells in the inguinal WAT of *Cd36*^CD81^ KO mice and littermate controls (Figure S7D). Without any stimuli, there was no difference in the proliferation between control and *Cd36*^CD81^ KO-derived CD81^+^ cells. However, linoleic-acid-induced cell proliferation was significantly attenuated in CD81^+^ cells from *Cd36*^CD81^ KO mice (Figure 6J). Second, *Cd36*^CD81^ KO mice and control mice were orally supplemented with linoleic acid for 14 days at 30°C (Figure S7E). We found that the number of Ki67^+^ CD81^+^ cells in the inguinal WAT of *Cd36*^CD81^ KO was lower than that of control mice following linoleic acid supplementation (Figure 6K). Attenuated CD81^+^ cell proliferation led to impaired beige fat biogenesis as the additive effect of cold and linoleic acid supplementation on beige fat biogenesis was blunted in *Cd36*^CD81^ KO mice (Figure 6L). Additionally, the inguinal WAT depots of *Cd36*^CD81^KO mice harbored fewer multilocular beige adipocytes than those of control mice (Figures 6M and S7F).

## DISCUSSION

**Figure 7.**
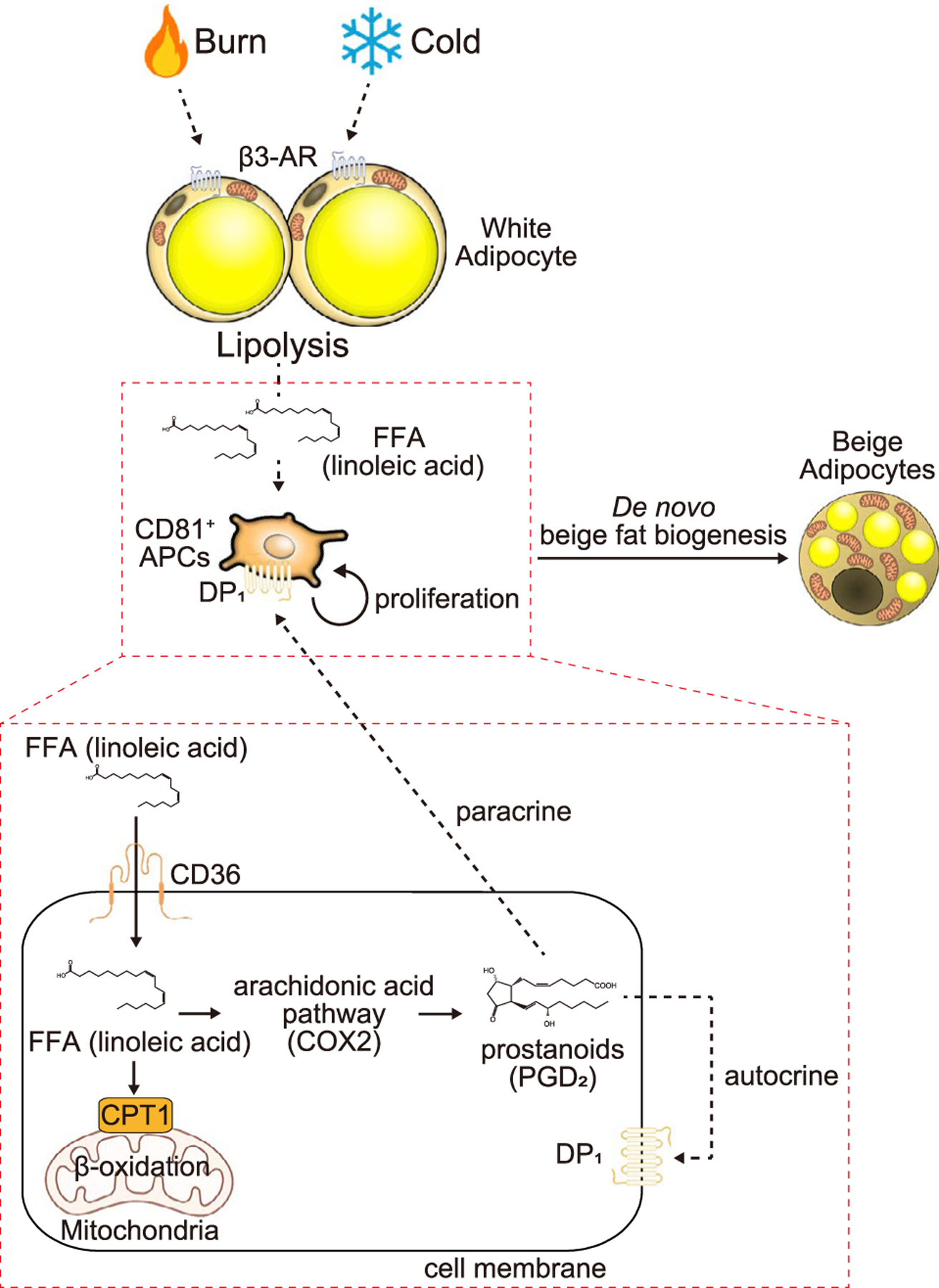
A model of *de novo* beige fat biogenesis Cold, β3-AR agonist, and burn injury trigger WAT lipolysis and the release of free fatty acids. CD81^+^ beige progenitor cells actively uptake linoleic acid through the plasma membrane fatty acid transporter CD36 for mitochondrial β-oxidation and also for the synthesis of arachidonic-acid-derived metabolites, including PGD_2_ via the Cox2 pathway. PGD_2_ stimulates CD81^+^ cell proliferation by paracrine or autocrine manner via the receptor DP1.

Our results propose the following model in which pathophysiological browning stimuli, cold acclimation, β3-AR activation, and burn injury promote *de novo* beige fat biogenesis (Figure 7). CD81^+^ progenitor cells in the subcutaneous WAT harbor cristae-rich mitochondria and actively uptake WAT lipolysis-derived fatty acids, particularly linoleic acid, through the plasma membrane transporter CD36. Linolic acid is utilized for mitochondrial β-oxidation and also for the synthesis of arachidonic acid-derived signaling entities, including PGD_2_, that stimulate CD81^+^ cell proliferation. Oral linoleic acid supplementation was sufficient to increase the beige progenitor cell pool even under a thermoneutral condition in a CD36-dependent manner. Notably, this mechanism also plays a vital role under a pathological condition, burn injury. This is in agreement with previous reports that blockade of lipolysis by inhibiting ATGL or hormone-sensitive lipase ameliorates cachexia-associated WAT loss and burn-injury-induced metabolic dysfunction in WAT.^41,42^ It is worth noting that serum concentration of linoleic acid was acutely elevated following burn injury, showing a positive correlation with clinical severities, such as hypermetabolic states and mortality.^43^ Thus, it is conceivable that inhibition of the linoleic acid pathway and/or prostanoid synthesis by the Cox2 pathway may be effective in attenuating burn injury-induced beige fat biogenesis and hypermetabolic state.

This work highlighted the critical role of mature adipocytes, i.e., lipolysis-derived signaling entities, for *de novo* beige fat biogenesis. This is also consistent with a recent work that mature-adipocyte-derived factors, such as β-hydroxybutyrate, that enhance beige adipogenesis.^44^ In humans, a β2-AR agonist, formoterol, stimulated human BAT thermogenesis.^45^ Given the stimulatory effect of β2-AR agonists on lipolysis in human adipose tissues,^46–48^ it is likely that chronic β2-AR activation also stimulates beige progenitor cell proliferation. Indeed, chronic β2-AR activation by formoterol was shown to promote beige fat biogenesis in mice.^49^

It is important to note that the role of fatty acids for beige progenitors is far more than merely fuel. Our lipidomics analysis identified several linoleic-acid-derived metabolites that await future studies. For instance, CD81^+^ cells contained high levels of 9-HETE and 12-HETE that were previously shown to bind steroid receptor coactivator-1 and PPARγ.^50,51^ 12-HETE is also released from BAT and stimulates glucose uptake in the BAT.^52^ 9-HODE, 13-HODE, and 15-keto PGE_2_ are known to act as ligands of PPARγ and stimulate its transcriptional activity.^53,54^ CD81^+^ cells also contained higher levels of EPA-derived metabolites, such as 18-hydroxyeicosapentaenoic acid (18-HEPE) and 12-HEPE, which inhibit the pro-inflammatory action of macrophages, thereby suppressing cardiac fibroblasts^55^ and atherosclerosis,^56^ respectively. These observations are intriguing, given the emerging role of beige fat in repressing adipose tissue fibrosis and inflammation.^44,57^ An important area of future research is to delineate how these lipids from beige progenitor cells contribute to adipose tissue remodeling.

### Limitations of the study

A report showed persistent nuclear translocation of the Cre-ERT2 protein in mouse adipose tissues even after 2 months of washout following tamoxifen treatment.^58^ Thus, we are aware of the limitation of the Cre-ER system for the study of adipose tissue biology, although our mouse studies examined phenotypic differences between KO mice versus littermate controls, both of which received tamoxifen. Another caveat is related to significant individual variations in the number of proliferative CD81^+^ cells in human subcutaneous WAT at a basal state. Besides, this study is limited to female subjects. Due to the limited number of subjects, this work could not delineate the relationship between the proliferation capacity of CD81^+^ cells and metabolic health, age, or any other factors, but our future study will interrogate the relationship and the underlying mechanisms. This is significant given the recent reports that low lipolytic activity in the subcutaneous WAT predicts future risks of impaired glucose homeostasis in women,^59^ and downregulation of β3-AR leads to adipocyte catecholamine resistance in obesity.^60^ Determining the degree to which oral supplementation of linoleic acid increases the beige progenitor cell pool in humans is also of paramount importance.

## STAR★METHODS

### RESOURCE AVAILABILITY

#### Lead contact

Further information and requests for resources and reagents should be directed to and will be fulfilled by the lead contact, Dr. Shingo Kajimura (skajimur@bidmc.harvard.edu).

#### Materials availability

Unique materials and reagents generated in this study are available upon request from the lead contact with a completed Materials Transfer Agreement.

#### Data and code availability

The RNA-seq data related to Figures 5A, 5E, 5F, and 6A have been deposited in NCBI’s Gene Expression Omnibus and are accessible through GEO Series accession number GSE201930.

### EXPERIMENTAL MODEL AND SUBJECT DETAILS

#### Animals

All animal experiments were performed according to procedures approved by the Institutional Animal Care and Use Committee for animal care and handling at the University of California, San Francisco (UCSF), Beth Israel Deaconess Medical Center, and the Sunnybrook Research Institute Animal Care Committee (for burn injury studies). C57BL/6J wild-type mice were obtained from the Jackson Laboratory (Stock No. 000664). For the generation of CD81^+^ cell-specific *Pparg* KO mice (*Cd81*-Cre^ERT2^; *Pparg*^flox/flox^), *Cd81*-Cre^ERT2^ mice were crossed with *Pparg*-floxed mice (Jackson Laboratory, Stock No. 004584). For the generation of CD81^+^ cell-specific *Cd36* KO mice (*Cd81*-Cre^ERT2^; *Cd36*^flox/flox^), *Cd81*-Cre^ERT2^ mice were crossed with *Cd36*-floxed mice (Jackson Laboratory, Stock No. 032276). For the generation of adipocyte-specific ATGL KO mice (*Adipoq*-Cre; *Pnpla2* ^flox/flox^), *Adipoq*-Cre mice were crossed with *Pnpla2*-floxed mice (Jackson Laboratory, Stock No. 024278). For the generation of *Cd81*-lineage reporter mice (*Cd81-Cre*^*ERT2*^; *Rosa26*-mTmG mice), *Cd81*-Cre^ERT2^ mice were crossed with *Rosa26*-mTmG mice (Jackson Laboratory, Stock No. 007576). These are all in C57BL/6J background. To induce Cre expression, tamoxifen at 2 mg in 100 μL corn oil per dose was administered intraperitoneally for 5 days.

Mice were maintained on a standard rodent chow at ambient temperature (22°C) under a 12 hr:12 hr light-dark cycle. Samples were obtained from male mice at 8–16 weeks, 20 weeks, or 30 weeks of age. For cold-exposure studies, mice were acclimated to 30°C for 2 weeks and exposed to 8°C for up to 3 days in a rodent incubator (Power Scientific, Inc. RIS33SD and RIS52SD). For the treatment with a β3-AR agonist, CL316,243 (Sigma-Aldrich) at 1 mg kg^−1^ body weight or vehicle was injected intraperitoneally for 3 consecutive days. For the treatment with a Cox2-inhibitor, Celecoxib (Sigma-Aldrich) at 15 mg kg^−1^ body weight or vehicle was administered intraperitoneally for 8 days.

#### Burn injury studies in mice

All mice were cared for in accordance with the Guide for Care and Use of Laboratory Animals, and all procedures were approved by the Sunnybrook Research Institute Animal Care Committee under AUP 467 (Toronto, ON, Canada). 10-weeks-old male C57BL/6 mice from Jackson Laboratories were housed individually at room temperature and kept on a 12 hr:12 hr light-dark cycle. Mice had ad libitum access to water and standard rodent chow. Following one week of acclimation, mice were divided into two weight-matched groups: sham and burn. Mice denoted burn were anesthetized with 2.5% isoflurane gas. Adequacy of the depth of anesthesia was assured by the absence of pedal withdrawal reflex. Mice were shaved along the dorsum and approximately 1cm^2^ on the abdomen and received buprenorphine (intraperitoneally; 0.05–0.1mg/kg × body mass) for pain management and Ringer’s lactate (SQ; 2–3mL) for resuscitation. As previously described, mice were inflicted with a 30%total body surface area full-thickness burn injury. This was achieved by immersing the dorsum in a 98°C water bath for 10 s and the ventral region for 2 s to avoid organ damage.^61^ Sham mice underwent similar procedures with the exception of the burn injury. All mice were placed individually within a warm, well-ventilated sterile cage following the procedure. Health, as determined by alertness, activity, food intake, posture, and hydration, was monitored twice daily throughout the duration of the study by both research and veterinarian staff. An additional dose of buprenorphine was given if mice showed signs of pain, such as a hunched posture or lethargy. Mice were also supplemented with wet food and Ringer’s lactate if they showed signs of dehydration. The concentrations of free fatty acid in plasma were measured with a Free Fatty Acid Quantifcation Kit (Abcam, Cambridge, MA), following the manufacturer’s instructions.

#### Human subjects

Subcutaneous adipose tissue was collected under Beth Israel Deaconess Medical Center Committee on Clinical Investigations IRB 2011P000079. Potential subjects were recruited in a consecutive fashion, as scheduling permitted, from the plastic surgery operating room rosters at Beth Israel Deaconess Medical Center. Subjects over the age of 18, who underwent elective plastic surgery procedures and free of other acute medical conditions, were included and provided written informed consent preoperatively. Excess adipose tissue from the surgical site was collected at the discretion of the surgeon during the normal course of the procedure. Subjects with a diagnosis of diabetes, or taking insulin-sensitizing medications such as thiazolidinediones or metformin, chromatin-modifying enzymes such as valproic acid, anti-retroviral medications, or drugs known to induce insulin resistance such as mTOR inhibitors or systemic steroid medications, were excluded. Age, gender, BMI of human subjects are listed in Table S1.

#### Linoleic acid supplementation *in vivo*

Male mice at 20 or 30 weeks old were given access to water bottles containing either vehicle (0.3% Xanthan gum in deionized water) or linoleic acid (1% volume in 0.3% Xanthan gum in deionized water) for two weeks. Xanthan gum solution was used to equalize the solution’s texture according to the published protocol used by several independent studies.^62–66^ The solutions were replaced every 24 h.

#### *Ex vivo* adipose tissue culture

Inguinal WAT from wild-type mice and Adipo-ATGL KO mice were cut into pieces weighing approximately 10 mg. For human subcutaneous WAT culture, a total of 120 mg of human adipose tissue biopsies were divided into 6 pieces, and each piece was cut into pieces weighing approximately 10 mg. Adipose tissue samples were quickly washed three times with Phosphate-buffered saline (PBS) and centrifuged for 1 min at 300 ×g at room temperature. Subsequently, adipose tissues were cultured overnight in DMEM/F12 medium containing 1%GlutaMAX-I and 10% FBS. After the preincubation, adipose tissue biopsies were washed three times with PBS and centrifuged for 1 min at 300 × g at room temperature (20–25°C). Then, adipose tissue samples were cultured with vehicle or CL316,243 at doses ranging from 0.1 μM to 1 μM in DMEM/F12 without FBS for 12 h (mice) or 24 h (human) for FACS analyses. To pharmacologically inhibit ATGL, Atglistatin (50 μM, Cayman) or vehicle (DMSO) was dissolved in DMEM/F12 containing 10% FBS.

#### Cell isolation and sorting

Stromal vascular fractions (SVFs) were isolated from the inguinal WAT depots of mice using Collagenase D (1.5 U ml^−1^) and Dispase II (2.5 U ml^−1^) following the procedure.^67^ MACS^®^ Non-Adipocyte Progenitor Depletion Cocktail for mice (130-106-639, Miltenyi Biotec) and MACS LS columns (Miltenyi Biotec) were used to deplete lineage^+^ (Lin^+^) cells. The following antibodies were used for the isolation of mouse CD81^+^ cells (Lin^−^: Sca1^+^: CD81^+^): Sca-1-PB (1:800, 108120, Biolegend) and CD81-APC (1:50, 104910, Biolegend) in autoMACS Rising Solution (Miltenyi Biotec) containing 0.5% BSA in the dark at 4°C for 15 min. For intracellular staining, cells were stained with the above antibodies and fixed in 4% PFA for 15 min. Subsequently, cells were incubated in autoMACS Rinsing Solution containing 0.5% BSA and 0.1% saponin for 15 min and stained with Ki67-PE antibody for 30 min (1:300, 151209, Biolegend). Cell population (%) was calculated as the frequency of parent. All the cells were isolated and analyzed using a FACS Aria II equipped with 100 mm nozzle diameter and CytoFLEX. FlowJo software (version 10.8.1) and CytExpert (version 2.4.0.28) were used for data analyses. For the quantification of CD36 protein expression, cells were stained with Sca-1-PB (1:800, 108120, Biolegend), CD81-APC (1:50, 104910, Biolegend), and CD36-PE (1:150, 102615, Biolegend) in autoMACS Rising Solution (Miltenyi Biotec) containing 0.5% BSA in the dark at 4°C for 15 min.

For human tissues, subcutaneous WAT depots were digested at 37°C using 2 mg ml^−1^ Collagenase Type II (Worthington) to isolate SVFs. The digests were washed with autoMACS Rinsing Solution containing 0.5% BSA and strained into fresh 15 mL plastic tubes using 100 µm filters to remove any undigested tissue, and the tubes were spun at 300 g for 10 min at 4°C. The pellets containing the SVF cells were resuspended in autoMACS Rinsing Solution containing 0.5% BSA, and strained into fresh 15 mL plastic tubes using 50 μm filters, and the tubes were spun at 300 g for 10 min at 4°C. The following antibodies were used for the isolation of human CD81^+^ cells (Lin^−^: PDGFRa^+^: CD81^+^): CD14-FITC (1:400, 301803, Biolegend), CD31-FITC (1:200, 303103, Biolegend), CD45-FITC (1:200, 304005, Biolegend), CD235a-FITC (1:500, 349103, Biolegend), PDGFRa (CD140a)-PerCP-Cy5.5 (1:200, 563575, BD Biosciences) and CD81-APC (1:500, 349510, Biolegend) in autoMACS Rising Solution containing 0.5% BSA in the dark at 4°C for 15 min. For intracellular staining, isolated SVFs were stained with the above antibodies and immediately fixed in 4% PFA for 15 min. Cells were incubated in autoMACS Rising Solution containing 0.5% BSA and 0.1% saponin for 15 min and stained with Ki67-PE antibody for 30 min (1:500, 350503, Biolegend). Cell population (%) was calculated as the frequency of parent.

#### Cell culture

Mouse SVFs cells were isolated from inguinal WAT depots by collagenase digestion following the protocol.^67^ Subsequently, CD81^−^ cells (Lin^−^: Sca1^+^: CD81^−^) and CD81^+^ cells (Lin^−^: Sca1^+^: CD81^+^) were isolated by using FACS Aria II and seeded onto non-coated plates. For adipocyte differentiation, immortalized SVFs from the inguinal WAT of C57BL/6J mice were differentiated in DMEM media containing an adipogenic cocktail (0.5 mM IBMX, 2 mg ml^−1^ dexamethasone, 850 nM insulin, 1 nM T3, 125 μM indomethacin with or without 1 μM rosiglitazone) for two days and subsequently in maintenance media containing 1 nM T3 and 850 nM insulin for another 4 days. Serum-free culture media were collected from differentiated adipocytes that were treated with CL316,243 or vehicle for 6 h. A part of the media was filtered and either heated at 95°C for 10 min or treated with chloroform/methanol to remove or extract fatty acids.^68^

### METHOD DETAILS

#### Cell proliferation assays

Inguinal WAT-derived primary CD81^+^ and CD81^−^ cells were incubated in DMEM/F12 media with the following fatty acids and chemical inhibitors. All the fatty acids used in the experiments were conjugated with fatty acid-free BSA (Sigma-Aldrich) in 2:1 molar ratio^69–71^ and incubated at doses ranging from 1 μM to 10 μM. The media were replaced every 48 h, and cell growth was monitored for four days. For the chemical inhibitors, Cox1 inhibitor (SC-560, 1 μM, Sigma-Aldrich), Cox2 inhibitor (NS-398, 1 μM, Sigma-Aldrich), Lox5 inhibitor (MK-886, 1 μM, Sigma-Aldrich), and Lox12 inhibitor (Baicalein, 1 μM, Sigma-Aldrich) or vehicle (DMSO) were dissolved in DMEM containing 10% FBS. For prostanoids, prostaglandin D_2_ at 1 μM and inhibitor for DP1 (MK-0524, 1 μM, Sigma-Aldrich) were dissolved in DMEM containing 10% FBS. For β-adrenoreceptor agonists, CL316,243, isoproterenol, or norepinephrine at 1 μM were used.

#### Cell cycle assays

Inguinal WAT-derived primary CD81^+^ cells were incubated with 10 μM EdU (Click-iT Plus EdU Flow Cytometry Assay Kit, Thermo Fisher Scientific, USA) for 1 h in DMEM/F12 media. Subsequently, cells were fixed according to the manufacturer’s instructions. FxCycle Violet Ready Flow Reagent was used to measure the total DNA content, following the manufacturer’s instructions. Cell population (%) was calculated as the frequency of parent. All the cells were analyzed using a FACS Aria II equipped with 100 mm nozzle diameter and CytoFLEX. FlowJo software (version 10.8.1) and CytExpert (version 2.4.0.28) were used for data analyses.

#### Lipidomics

Liquid chromatography with tandem mass spectrometry (LC-MS/MS) analysis was performed for targeted eicosanoids profiling of CD81^−^ cells and CD81^+^ cells in the Stable Isotope and Metabolomics Core Facility of the Albert Einstein College of Medicine. Cell pellets were extracted with phosphate buffer solution with internal standards. The extraction was loaded onto a StrataX SPE column. Eicosanoids were eluted with methanol. The samples were dried under gentle nitrogen flow. The dried samples were reconstituted into 100 μl of methanol, and pending LC injection. The analytes were separated on a BEH shield RP18 column and analyzed with ABsciex 6500+ QTrap instrument in a targeted Multiple Reaction Monitoring (MRM) mode. A pooled quality control (QC) sample was added to the sample list. This QC sample was injected six times for coefficient of variation (CV) calculation for data quality control. The eicosanoids with CVs of less than 30% were selected for the statistical analysis.

For the quantification of linoleic acid, LC/MS studies were performed at the Beth Israel Deaconess Mass Spectrometry Facility. Non-polar lipid samples were resuspended in 35 μL of 1:1 LC/MS grade isopropanol:methanol prior to LC-MS/MS analysis, 5 μL were injected. A Cadenza 150 mm × 2 mm3 μm C18 column (Imtakt) heated to 40°C at 240 μL/min was used with a 1100 quaternary pump HPLC with room temperature autosampler (Agilent). Lipids were eluted over 22 min. gradient from 32% B buffer (90% IPA/10% ACN/10 mM ammonium formate/0.1 formic acid) to 97% B. A buffer consisted of 59.9% ACN/40% water/10 mM ammonium formate/0.1% formic acid. Lipids were analyzed using a high-resolution hybrid QExactive HF Orbitrap mass spectrometer (Thermo Fisher Scientific) in DDA mode (Top 8) using positive/negative ion polarity switching. DDA data were acquired from m/z 225-1450 in MS1 mode, and the resolution was set to 70,000 for MS1 and 35,000 for MS2. MS1 and MS2 target values were set to 5e5 and 1e6, respectively. Lipidomic data were analyzed for both identification and relative quantification of linoleic acid and its derivatives using Scaffold Elements 3.0 software (Proteome Software) using the NIST v20, HMDB and LipidMaps databases and isolating the searches to ~280 +/− 50 m/z for linoleic acid detection.

#### RNA-seq analyses

Total RNA was isolated from primary inguinal WAT-derived CD81^+^ and CD81^−^ cells from wild-type C57BL/6J mice at 10 weeks old using RNeasy Micro Kit (QIAGEN). Extracted RNA samples were quantified using Qubit 2.0 Fluorometer (Life Technologies, Carlsbad, CA, USA), and RNA integrity was checked using Agilent TapeStation 4200 (Agilent Technologies, Palo Alto, CA, USA). RNA sequencing libraries were prepared using the NEBNext Ultra II RNA Library Prep Kit for Illumina following the manufacturer’s instructions (NEB, Ipswich, MA, USA). Briefly, mRNAs were first enriched with Oligo(dT) beads. Enriched mRNAs were fragmented for 15 min at 94°C. First-strand and second-strand cDNAs were subsequently synthesized. cDNA fragments were end-repaired and adenylated at 3’ends, and universal adapters were ligated to cDNA fragments, followed by index addition and library enrichment by limited-cycle PCR. The sequencing libraries were validated on the Agilent TapeStation (Agilent Technologies, Palo Alto, CA, USA) and quantified by using Qubit 2.0 Fluorometer (Invitrogen, Carlsbad, CA) as well as by quantitative PCR (KAPA Biosystems, Wilmington, MA, USA). The sequencing libraries were clustered on 2 flowcell lanes. After clustering, the flowcell was loaded on the Illumina HiSeq instrument (4000 or equivalent) according to the manufacturer’s instructions. The samples were sequenced using a 2×150bp Paired-End (PE) configuration. Image analysis and base calling were conducted by the HiSeq Control Software (HCS). Raw sequence data (.bcl files) generated from Illumina HiSeq was converted into fastq files and de-multiplexed using Illumina’s bcl2fastq 2.17 software. One mismatch was allowed for index sequence identification.

#### Bioinformatics

Fastq files were pseudoaligned against the mouse transcriptome by Kallisto (version 0.46.1) with default parameters.^72^ All downstream analyses were performed using R. Transcript-level raw counts from Kallisto were imported into R using the Bioconductor package tximport (version 1.12.3), and the expression levels of each gene were estimated. Differential expression analysis between the two groups was performed using the DESeq2 (1.24.0), which generated log_2_(‒fold change) and adjusted *p*-value. Raw fastq files were deposited to the National Center for Biotechnology Information Gene Expression Omnibus database under accession number GSE201930.

#### qRT-PCR

Total RNA was extracted from tissue using TRIzol reagents (Thermo Fisher) and purified using the Direct-zol RNA Miniprep Kits (Zymo research). Complementary DNA was synthesized using the iScript cDNA Synthesis Kit (Bio-Rad Laboratories) according to the provided protocol. Quantitative RT-PCR was performed using a QuantStudio^™^ 6 (Life technologies). The relative mRNA expression in each sample was normalized to the TATA-binding protein or 36B4 by determining by the ΔΔ Ct method and normalized to an internal calibrator specific to each gene using the formula 2^−ΔΔCT^. The primer sequences used in the study are provided in Table S2.

#### Tissue histology and immunohistochemistry

For hematoxylin and eosin (H&E) staining, tissues of mice were fixed in 4% paraformaldehyde (PFA) overnight at 4°C, followed by dehydration in 70% ethanol. After dehydration, tissues were embedded in paraffin, sectioned at a thickness of 5 μm, and stained with H&E following the standard protocol. Images were acquired using a Zeiss AxioImager M1 (Carl Zeiss). For immunostaining, paraffin-embedded tissues were deparaffinized twice in xylene and subsequently rehydrated. After heat-induced epitope retrieval using target retrieval solution (Dako), the tissues were blocked in PBS containing 10% goat serum with 0.1% Tween 20 for 60 min. After washing in PBS, slides were incubated with rabbit anti-UCP-1(1:200, ab10983, Abcam), rabbit anti-Ki67(1:200, 12202, Cell Signaling), and goat anti-GFP (1:200, NB100-1678, Novus Biologicals) antibody overnight at 4°C, followed by incubation with Alexa Fluor 488 antibody (1:500, Thermo Fisher) for GFP, and Alexa Fluor 647 antibody (1:500, Thermo Fisher) for UCP1, Ki67 for 60 min at room temperature. After washing, the sections were stained with Hoechst 33342 for nuclei and mounted with a mounting medium (Cytoseal 60, Thermo Scientific). Images of tissue samples were captured using the Zeiss AxioImager M1 (Carl Zeiss) and analyzed using the AxioVision software (Version 4.8). The number of GFP^+^ Ki67^+^ cells was evaluated in 8 randomly selected fields from each sample.

#### Immunoblotting

Tissues were lysed in RIPA lysis and extraction buffer (Thermo Fisher) and protease inhibitors (Roche). Total protein lysates were boiled with Laemmli sample buffer containing 355 mMb-mercaptoethanol, loaded on a 12%, or 4%–20% SDS-PAGE. Subsequently, separated proteins were transferred onto PVDF membranes. PVDF membrane blots were blocked in Blocking Buffer (Bio-rad) for 5 min at room temperature and incubated overnight at 4°C with rabbit anti-UCP-1 (1:1,000, ab10983, Abcam) or anti-β-actin (1:20,000, A3854, Sigma-Aldrich). Mitochondrial proteins were detected using Total OXPHOS Rodent WB Antibody Cocktail (1:2,000, ab110413, Abcam). Anti-rabbit IgG (ab6721, Abcam) was used as a second antibody for UCP1. Anti-mouse IgG (31430, Thermo Fisher) was used as a secondary for Total OXPHOS Rodent WB Antibody Cocktail.

#### Metabolic analysis in mice

*Pparg*^CD81^ KO mice and littermate control mice were exposed to 8°C for 5 days before transferring to metabolic cages at 30°C. Mice received intraperitoneal (i.p.) injection of β3-AR agonist, CL316,243 (Sigma; 0.5 mg per kg body weight) during the metabolic analysis. Whole-body energy expenditure (VO_2_), food intake, and locomotor activity (beam break counts) were monitored and recorded using the Comprehensive Laboratory Animal Monitoring System (CLAMS, Columbus Instruments). Obtained indirect calorimetry data were analyzed by CaIR-ANCOVA (https://calrapp.org/).

#### Oxygen consumption assays

Cellular OCR was measured using the Seahorse XFe Extracellular Flux Analyzer (Agilent). Inguinal WAT-derived CD81^−^ cells and CD81^+^ cells were seeded at 40,000 cells per well in a Seahorse XF 24-well plate. After overnight culture, cells were maintained in the XF assay medium supplemented with 1 mM sodium pyruvate, 2 mM glutamine, and 10 mM glucose. For the measurement of uncoupled respiration, cells were treated with 5 μM oligomycin, followed by carbonyl cyanide 4-(trifluoromethoxy) phenylhydrazone (FCCP, 5 μM), and antimycin A (5 μM). For linoleic acid experiments, BSA-conjugated linoleic acid at 5 μM and/or a CPT inhibitor (etomoxir) at 50 μM were added to the XF assay medium supplemented with 1 mM sodium pyruvate, 2 mM glutamine, and 10 mM glucose.

#### Electron microscopy

Inguinal WAT-derived CD81^−^ cells and CD81^+^ cells were immersion fixed in 2.5% glutaraldehyde (Electron Microscopy Sciences Hatfield, PA) and 2% formaldehyde (Electron Microscopy Sciences), in 0.1 M phosphate buffer (Sigma-Aldrich, St. Louis, MO) pH 7.4 for 1hr at room temperature and then embedded in low gelling temperature sea plaque agarose (Cambrex Biosciences, Rockland, ME). Blocks of agar-containing cells were fixed overnight in the original fixative, washed with 0.1 M phosphate buffer, and then post-fixed for 1hr at 4°C in 1% osmium tetroxide (Electron Microscopy Sciences) containing 0.03 g/4 ml potassium ferrocyanide (Sigma-Aldrich) in 0.1M phosphate buffer. Cells were washed in DI water and incubated in 2% aqueous uranyl acetate (Electron Microscopy Sciences) overnight at 4°C. The following day, cells were washed with DI water and then dehydrated at 4°C in a graded ethanol series. The agar blocks were then brought to room temperature and dehydrated with 100% ethanol (Sigma-Aldrich) followed by propylene oxide (Electron Microscopy Sciences). Infiltration in LX112 resin (Ladd Research Industries, Williston, VT), was followed by embedding in flat bottom Beem capsules (Electron Microscopy Sciences). The resulting blocks were sectioned using a Leica Ultracut E ultramicrotome, and sections were placed on formvar (Electron Microscopy Sciences) and carbon-coated grids. The sections were contrast stained with 2% uranyl acetate followed by lead citrate (Sigma-Aldrich), and imaged in a JEOL 1400 transmission electron microscope (JEOL, Peabody, MA) equipped with a Gatan Orius SC1000 digital CCD camera (Gatan, Pleasanton, CA).

#### Glucose tolerance and insulin tolerance tests

Male *Pparg*^CD81^ KO and the littermate control mice at 8 weeks old were fed a high-fat diet (HFD, 60% fat, D12492, Research Diets) at 22 °C. Body weight was measured every week. For glucose tolerance tests, mice on a HFD for 10 weeks and fasted for 6 h from 9:00 to 15:00, were administered glucose intraperitoneally (1.0 g kg^−1^ body weight). For insulin tolerance tests, mice on a HFD for 9 weeks and fasted for 3 h from 9:00 to 12:00, were injected intraperitoneally with insulin (1 U kg^−1^ body weight). Blood samples were collected before injection, and glucose levels were measured using blood glucose test strips (Freestyle Lite).

### QUANTIFICATION AND STATISTICAL ANALYSIS

#### Statistics

Statistical analyses were performed using statistical software (JMP 12.0.1, SAS, and Prism 8, GraphPad). All data were represented as mean ± SEM, except where noted. Unpaired Student’s t test was used for two-group comparisons. One-way ANOVA followed by the Tukey-Kramer’s post hoc test was used for multiple group comparisons. Multilevel analysis was used for human subcutaneous WAT results. Two-way repeated-measures ANOVA was used for data on oxygen consumption, cell proliferation assays, body weight, whole-body energy expenditure, GTT, and ITT. *p* <0.05 was considered significant in all the experiments. The statistical parameters and the number of mice used per experiment are found in the figure legends.

## Supplementary Material

MMC1

## Figures and Tables

**Table:** KEY RESOURCES TABLE

REAGENT or RESOURCE	SOURCE	IDENTIFIER
Antibodies		

Anti-UCP1 antibody	abcam	Cat# ab10983; RRID: AB_2241462
Anti-Ki67 antibody	Cell Signaling Technology	Cat# 12202; RRID: AB_2620142
Anti-GFP antibody	Novus Biologicals	Cat# NB100-1678; RRID: AB_10002630
Anti-β-actin antibody	Sigma-Aldrich	Cat# A3854; RRID: AB_262011
Pacific Blue^™^ anti-mouse Ly-6A/E (Sca-1) Antibody	Biolegend	Cat# 108120; RRID: AB_493273
APC anti-mouse/rat CD81 Antibody	Biolegend	Cat# 104910; RRID: AB_2562996
PE anti-mouse/human Ki-67 Antibody	Biolegend	Cat# 151209; RRID: AB_2716014
FITC anti-human CD14 Antibody	Biolegend	Cat# 301803; RRID: AB_314185
FITC anti-human CD31 Antibody	Biolegend	Cat# 303103; RRID: AB_314329
FITC anti-human CD45 Antibody	Biolegend	Cat# 304005; RRID: AB_314393
FITC anti-human CD235a (Glycophorin A) Antibody	Biolegend	Cat# 349103; RRID: AB_10612923
PerCP-Cy^™^5.5 Mouse Anti-Human CD140a	BD	Cat# 563575; RRID: AB_2738286
APC anti-human CD81 (TAPA-1) Antibody	Biolegend	Cat# 349510; RRID: AB_2564021
PE anti-human Ki-67 Antibody	Biolegend	Cat# 350503; RRID: AB_10660818
Total OXPHOS Rodent WB Antibody Cocktail	abcam	Cat# ab110413; RRID: AB_2629281
Goat anti-Mouse IgG (H+L) Secondary Antibody, HRP	Thermo Fisher Scientific	Cat# 31430; RRID: AB_10960845
Goat Anti-Rabbit IgG H&L (HRP)	abcam	Cat# ab6721; RRID: AB_955447

Biological samples		

Human tissue samples	This paper	N/A

Chemicals, peptides, and recombinant proteins		

Advanced DMEM/F-12	Gibco	Cat# 12634010
Antimycin A from Streptomyces sp.	Sigma-Aldrich	Cat# A8674
Atglistatin	Cayman	Cat# 15284
autoMACS Rinsing Solution	Miltenyi	Cat# 130-091-222
Baicalein	Sigma-Aldrich	Cat# 196322
Bovine Serum Albumin	Sigma-Aldrich	Cat# A8806
Celecoxib	Sigma-Aldrich	Cat# SML3031
CL316,243	Sigma-Aldrich	Cat# C5976
Collagen I, Rat Tail	Corning	Cat# 354236
Collagenase D	Roche	Cat# 11088882001
cOmplete^™^, EDTA-free Protease Inhibitor Cocktail	Roche	Cat# 11873580001
Corn oil	Sigma-Aldrich	Cat# C8267
Dexamethasone	Sigma-Aldrich	Cat# D4902
Dispase II	Roche	Cat# 04942078001
DMEM	Gibco	Cat# 11966025
EveryBlot Blocking Buffer	Bio-rad	Cat# 12010020
Fetal Bovine Serum	ATLANTA biologicals	Cat# S11550
Formaldehyde	Thermo Fisher Scientific	Cat# 50-980-495
Glucose solution	Thermo Fisher Scientific	Cat# A2494001
Glutamax-I	Thermo Fisher Scientific	Cat# 35-050-061
Glutaraldehyde	Thermo Fisher Scientific	Cat# 50-259-41
Indomethacin	Sigma-Aldrich	Cat# I8280
Insulin	Sigma-Aldrich	Cat# I6634
Isobutylmethylxanthine (IBMX)	Sigma-Aldrich	Cat# I5879
(−)-Isoproterenol hydrochloride	Sigma-Aldrich	Cat# I6504
Lead citrate	Sigma-Aldrich	Cat# 15326
Linoleic acid	Thermo Fisher Scientific	Cat# AC215041000
Linoleic acid	Nu-Chek Prep	Cat# U-59-A
L-(−)-Norepinephrine(+)-bitartrate salt monohydrate	Sigma-Aldrich	Cat# A9512
LX112 resin	Thermo Fisher Scientific	Cat# NC9925769
MACS LS columns	Miltenyi	Cat# 130-042-401
MK-0524	Cayman	Cat# 10009835
MK-886	Sigma-Aldrich	Cat# 475889
Myristic acid	Nu-Chek Prep	Cat# N-14-A
Non-Adipogenic Progenitor Depletion Cocktail	Miltenyi	Cat# 130-106-639
Oleic acid	Nu-Chek Prep	Cat# U-46-A
Oligomycin	Cell Signaling Technology	Cat# 9996
Osmium Tetroxide	Thermo Fisher Scientific	Cat# 50-275-80
Palmitic acid	Nu-Chek Prep	Cat# N-16-A
Palmitoleic acid	Nu-Chek Prep	Cat# U-40-A
Paraformaldehyde	Santa Cruz Biotechnology	Cat# SC281692
PBS	Gibco	Cat# 10010023
Phenylhydrazone	Sigma-Aldrich	Cat# C2920
Phosphate buffer solution	Thermo Fisher Scientific	Cat# P5244
Potassium ferricyanide	Sigma-Aldrich	Cat# 702587
Propylene oxide	Thermo Fisher Scientific	Cat# 50-281-87
Prostaglandin D_2_	Cayman	Cat# 12010
RIPA Lysis and Extraction Buffer	Thermo Fisher Scientific	Cat# 89900
Rosiglitazone	Cayman	Cat# 71740
SeaPlaque Agarose	Cambrex Biosciences	Cat# 50101
Saponin	Sigma-Aldrich	Cat# 47036
SC-560	Sigma-Aldrich	Cat# 565610
Sodium Pyruvate	Cell Culture Facility-UCSF	Cat# CCFGE001
Stearic acid	Nu-Chek Prep	Cat# N-18-A
Sterile BSA	Sigma-Aldrich	Cat# A1595
Tamoxifen	Sigma-Aldrich	Cat# T5648
Target Retrieval Solution	Dako	Cat# S1699
Uranyl acetate	Thermo Fisher Scientific	Cat# NC1085517
Xanthan gum	Sigma-Aldrich	Cat# G1253
XF assay medium	Agilent	Cat# 102365-100
XF calibrant solution	Agilent	Cat# 100840-000
0.05% Trypsin	Corning	Cat# MT25052CI

Critical commercial assays		

Click-iT^™^ Plus EdU Flow Cytometry Assay Kits	Thermo Fisher Scientific	Cat# C10632
Direct-zol RNA Miniprep Kits	Zymo research	Cat# R2052
Free Fatty Acid Quantifcation Kit	abcam	Cat# ab65341
FxCycle^™^ Violet Ready Flow^™^ Reagent	Thermo Fisher Scientific	Cat# R37166
iscript reverse transcription supermix for rt-qPCR	Bio-rad	Cat# 1708841
iTaq Universal SYBR Green Supermix	Bio-rad	Cat# 1725125
NEBNext Ultra II RNA Library Prep Kit for Illumina	New England Biolabs	Cat# E7770
Pierce^™^ BCA Protein Assay Kit	Thermo Fisher Scientific	Cat# 23225
Rat/Mouse Insulin ELISA	Sigma-Aldrich	Cat# EZRMI
RNeasy Micro Kit	Qiagen	Cat# 74034
XFe24 FluxPak	Agilent	Cat# 102340-100
Seahorse XF24 Islet Capture Microplates	Agilent	Cat# 101122-100

Deposited data		

RNA-seq dataset	This paper	GEO Accession viewer: GSE201930

Experimental models: Cell lines		

Immortalized inguinal white adipocyte	Shinoda et al., 2015^73^	N/A

Experimental models: Organisms/strains		

Mouse: C57BL6J mice	The Jackson Laboratory	000664
Mouse: *Cd81*-Cre^ERT2^ mice	This paper	N/A
Mouse: *Pparg*^loxP^ mice	The Jackson Laboratory	004584
Mouse: Cd36^l^oxP mice	The Jackson Laboratory	032276
Mouse: *Adipoq*-Cre mice	Lee et al., 2013^74^	N/A
Mouse: *Pnpla2*^loxP^ mice	The Jackson Laboratory	024278
Mouse: *Rosa26*-mTmG mice	The Jackson Laboratory	007576

Oligonucleotides		

A full list of qPCR primers in TableS2	This paper	N/A

Software and algorithms		

Kallisto Version 0.46.1	Pachter Lab	https://pachterlab.github.io/kallisto/
Tximport Version 1.12.3	Bioconductor	http://bioconductor.org/packages/release/bioc/html/tximport.html
DESeq2 Version 1.24.0	Bioconductor	https://bioconductor.org/packages/release/bioc/html/DESeq2.html
Metascape pathway analysis	Zhou et al., 2019^75^	http://metascape.org/gp/index.html#/main/step1
Scaffold Elements 3.0	Proteome Software	https://www.proteomesoftware.com/products/scaffold-elements
CaIR-ANCOVA	Banks Lab	https://calrapp.org/
FlowJosoftware Version 10.8.1	BD	https://www.flowjo.com/
CytExpert Version 2.4.0.28	Beckman Coulter	https://www.beckman.com/flowcytometry/research-flow-cytometers/cytoflex/release-notes
GraphPad Prism 8	GraphPad Software	https://www.graphpad.com/scientificsoftware/prism/
JMP 12.0.1	SAS	https://www.jmp.com/en_us/software/data-analysis-software.html

Other		

High Fat Diet	Research Diets	Cat# D12492

## Highlights

- WAT lipolysis triggers beige adipocyte progenitor cell (APC) proliferation following cold and burn injury
- Beige APCs utilize linoleic acid for mitochondrial oxidation and prostanoid synthesis
- Linoleic acid supplementation sufficiently stimulates beige APC proliferation
- CD36 is required for beige APC proliferation in response to cold and linoleic acid
