# Supplementary material for: Lipolysis-derived linoleic acid drives beige fat progenitor cell proliferation
Source: Dev Cell. Author manuscript; Available in PMC 2023 Jan 25. (PMC9875052; doi:10.1016/j.devcel.2022.11.007)
Supplement: MMC1 [file NIHMS1853377-supplement-MMC1.pdf]

Developmental Cell, Volume *57*

Supplemental information

Lipolysis-derived linoleic acid drives beige fat progenitor cell proliferation

Ichitaro Abe, Yasuo Oguri, Anthony R.P. Verkerke, Lauar B. Monteiro, Carly M. Knuth, Christopher Auger, Yunping Qiu, Gregory P. Westcott, Saverio Cinti, Kosaku Shinoda, Marc G. Jeschke, and Shingo Kajimura

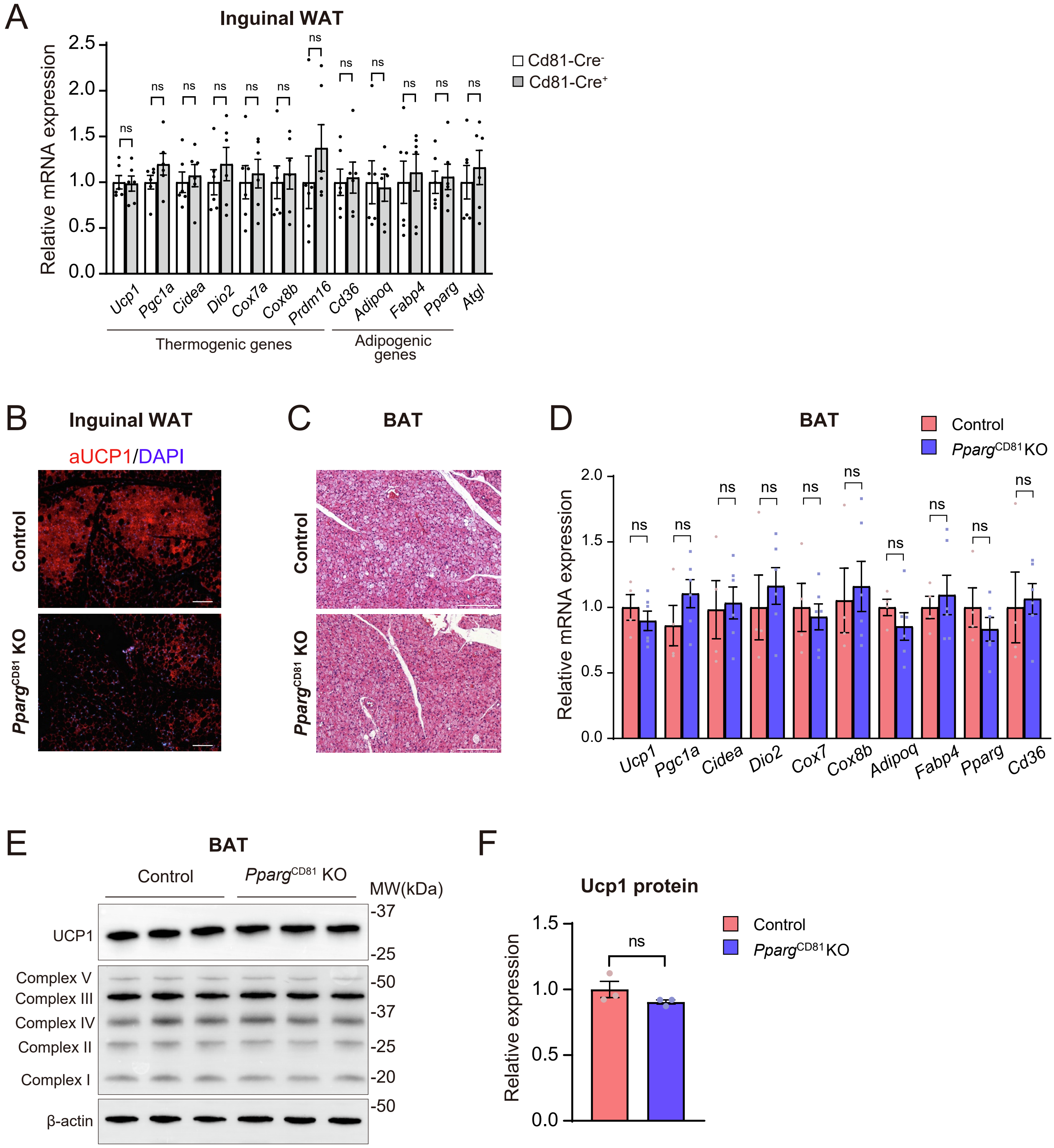

Supplemental Figure 1. (related to Figure 1): CD81 Is Dispensable for Brown Adipocyte Biogenesis in interscapular BAT.

A. Relative mRNA levels of indicated genes in the inguinal WAT of *Cd81*-Cre$^{ERT2/-}$ and littermate control (wild-type) mice following cold exposure at 8°C for 3 days. $n = 6$ for both groups, biologically independent mice.

B. Representative immunofluorescent staining for UCP1 (Red)/DAPI (Blue) in the inguinal WAT of *Pparg*^{CD81} KO and control mice at 8°C for 3 days. Scale bar, 100 μm.

C. Representative H&E staining in the interscapular BAT of *Pparg*^{CD81} KO and control mice at 8°C for 3 days. Scale bar, 100 μm.

D. Relative mRNA levels of indicated genes in the interscapular BAT of *Pparg*^{CD81} KO mice and littermate controls at 8°C. $n = 6$ for *Pparg*^{CD81} KO mice, $n = 4$ for controls.

E. Immunoblotting of UCP1 and indicated mitochondrial proteins in BAT of mice in (C). n = 3. Molecular weight (kDa) is shown on the right.

F. Immunoblotting-based quantification of UCP1 protein levels in (E). $n = 3$.

A, D, F. Data are mean ± SEM.; ns, not significant, by two-tailed unpaired Student's *t*-test.

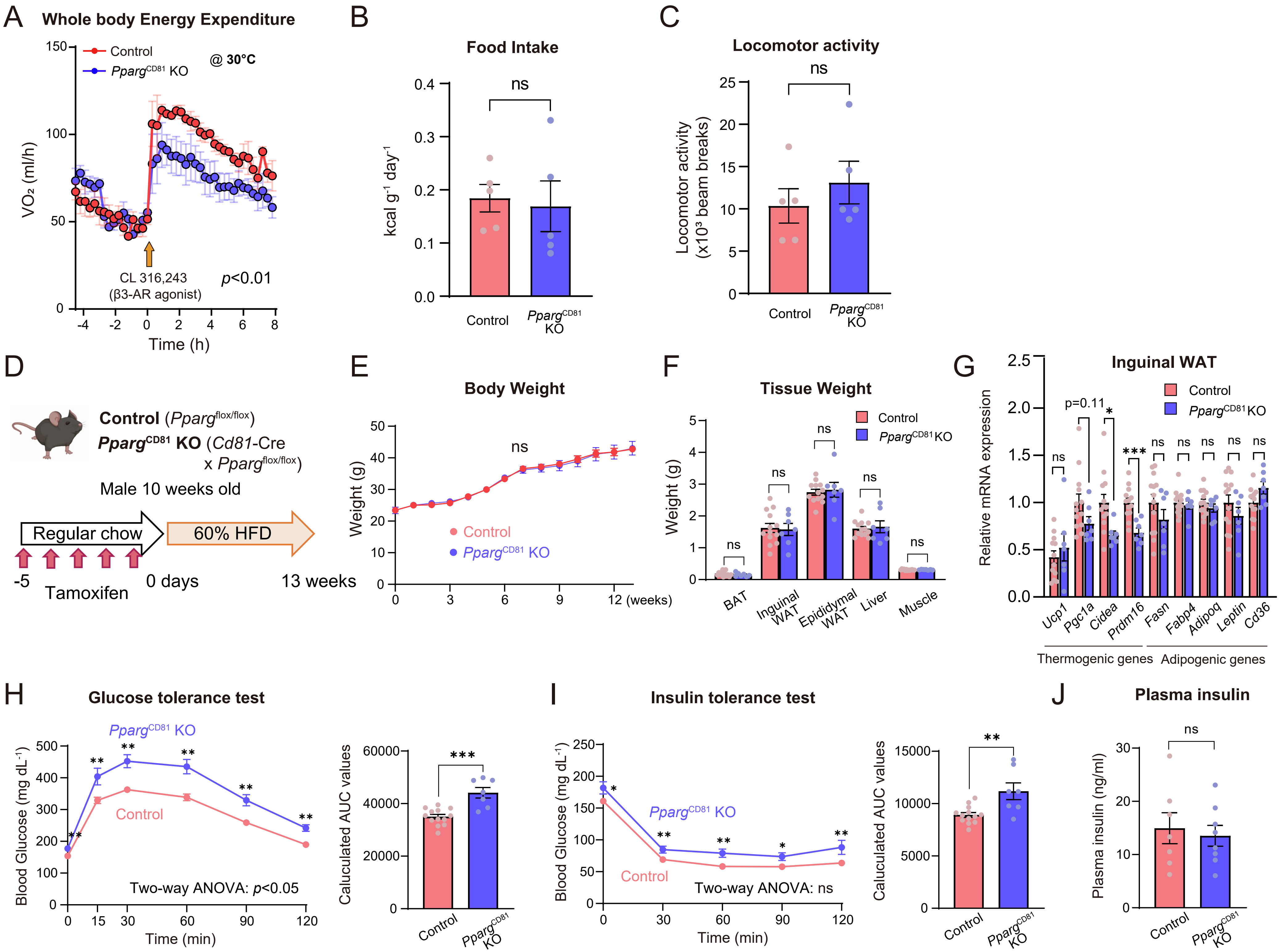

A Whole body Energy Expenditure
Control
Pparg CD81 KO
@ 30°C
VO2 (ml/h)
CL 316,243
(β3-AR agonist)
p<0.01
Time (h)
B Food Intake
kcal g-1 day-1
ns
Control
Pparg CD81 KO
C Locomotor activity
Locomotor activity (x10³ beam breaks)
ns
Control
Pparg CD81 KO
D
Control (Pparg flox/flox)
Pparg CD81 KO (Cd81-Cre x Pparg flox/flox)
Male 10 weeks old
Regular chow
60% HFD
-5
0 days
13 weeks
Tamoxifen
E Body Weight
Weight (g)
ns
Control
Pparg CD81 KO
12 (weeks)
F Tissue Weight
Weight (g)
Control
Pparg CD81 KO
ns
BAT
Inguinal WAT
Epididymal WAT
Liver
Muscle
G Inguinal WAT
Relative mRNA expression
Control
Pparg CD81 KO
p=0.11
*
***
ns
Ucp1
Pgc1a
Cidea
Prdm16
Fasn
Fabp4
Adipoq
Leptin
Cd36
Thermogenic genes
Adipogenic genes
H Glucose tolerance test
Blood Glucose (mg dL-1)
Pparg CD81 KO
Control
Two-way ANOVA: p<0.05
Time (min)
Caluculated AUC values
***
I Insulin tolerance test
Blood Glucose (mg dL-1)
Pparg CD81 KO
Control
Two-way ANOVA: ns
Time (min)
Caluculated AUC values
**
J Plasma insulin
Plasma insulin (ng/ml)
ns

Supplemental Figure 2. (related to Figure 1): Metabolic Phenotype of *Pparg*$^{\mathbf{CD81}}$ KO Mice.

A. Indirect calorimetric measurements (VO_2, oxygen consumption rate, ml min^{-1}) of *Pparg*^{CD81} KO and littermate control mice at 30 °C. The mice received i.p. injection of CL316,243 at 0.5 mg per kg^{-1} (orange arrow). n = 5.

B. Quantification of food intake in (A).

C. Quantification of locomotor activity in (A).

D. Schematic illustration of high-fat diet experiment. $CD81^{+}$ cell-specific *Pparg* knockout mice (*Pparg*^{CD81} KO, *Cd81*-Cre^{ERT2}; *Pparg*$^{flox/flox}$) and littermate controls (*Pparg*$^{flox/flox}$) received tamoxifen for 5 days and fed on a high-fat diet (HFD, 60% fat) for 13 weeks at 22°C. $n = 7$ for *Pparg*^{CD81} KO mice, $n = 13$ for controls.

E. Changes in body-weight of mice in (D).

F. Tissue weight of mice in (D).

G. Relative mRNA levels of indicated genes in the inguinal WAT of mice in (D).

H. Left: Glucose tolerance test in *Pparg*^{CD81} KO mice and littermate controls at 10 weeks of HFD. After 6 hours of fasting, mice received i.p. injection of glucose at of 1.0g kg^{-1} body-weight. n = 7 for *Pparg*^{CD81} KO mice, n = 13 for controls.

I. Left: Insulin tolerance test in *Pparg*^{CD81} KO mice and littermate controls at 9 weeks of HFD. Mice were fasted for 3 hours and subsequently received intra peritoneal injection of insulin at 1 U kg^{-1} body-weight. n = 7 for *Pparg*^{CD81} KO mice, n = 13 for controls.

J. Plasma insulin levels under fasted conditions in *Pparg*^{CD81} KO mice and littermate controls at 10 weeks of HFD. After 6 hours of fasting, blood samples were collected. $n = 7$ for *Pparg*^{CD81} KO mice, $n = 13$ for controls.

A, E. ns, not significant, by two-way repeated-measures ANOVA followed by two-tailed unpaired Student's *t*-test. **B, C, F, G, J.** Data are mean ± SEM.; * $p < 0.05$, *** $p < 0.001$, ns, not significant, by two-tailed unpaired Student's *t*-test. **H, I.** Data are mean ± SEM.; * $p < 0.05$, ** $p < 0.01$ by two-tailed unpaired Student's *t*-test. Area under the curve (AUC) was calculated by Prism software. ** $p < 0.01$, *** $p < 0.001$, by unpaired Student's *t*-test.

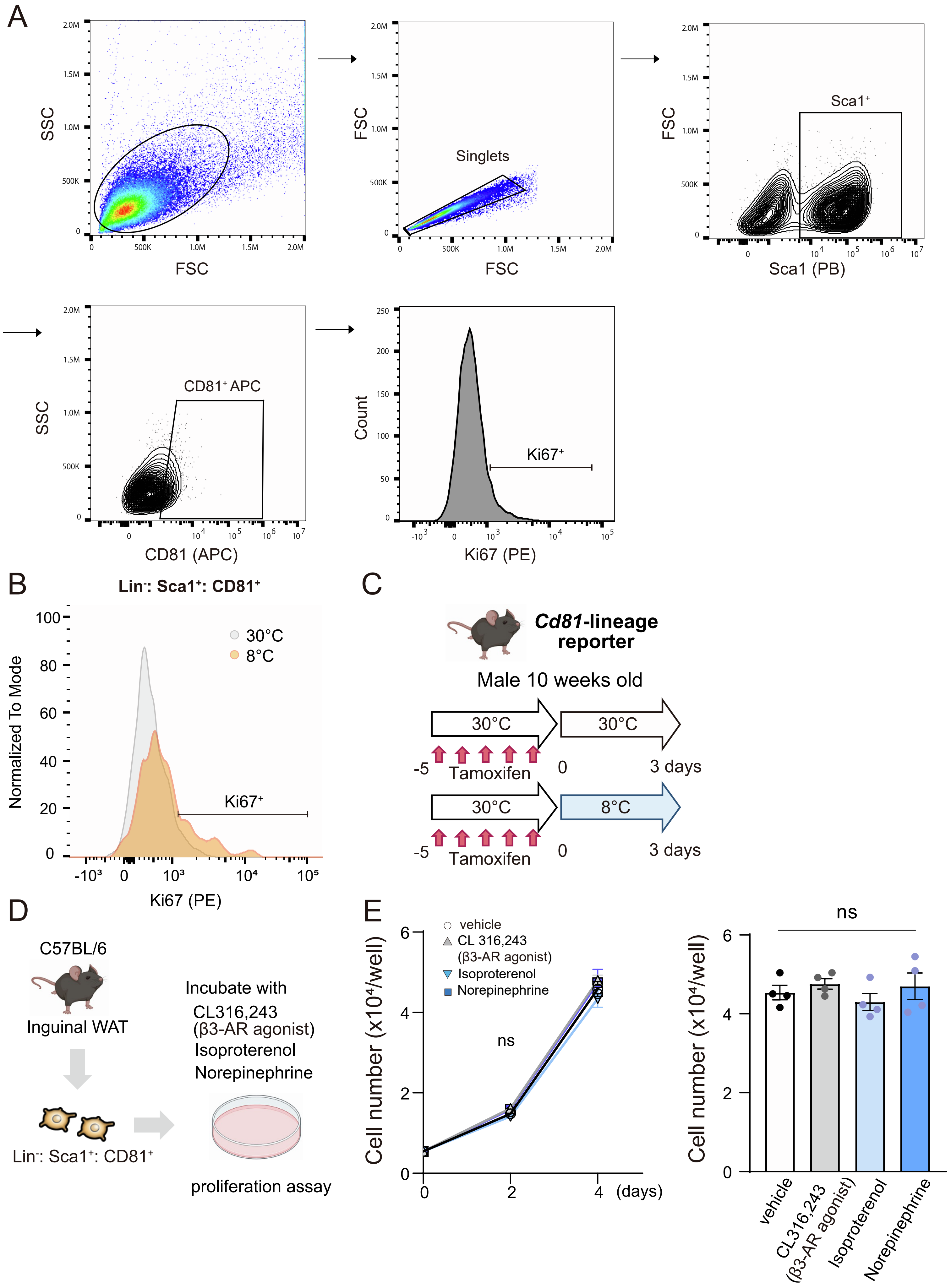

A
SSC
FSC
FSC
Singlets
FSC
Sca1⁺
Sca1 (PB)
SSC
CD81⁺ APC
CD81 (APC)
Count
Ki67⁺
Ki67 (PE)
B
Lin⁻: Sca1⁺: CD81⁺
Normalized To Mode
30°C
8°C
Ki67⁺
Ki67 (PE)
C
Cd81-lineage reporter
Male 10 weeks old
30°C
30°C
-5
Tamoxifen
0
3 days
30°C
8°C
-5
Tamoxifen
0
3 days
D
C57BL/6
Inguinal WAT
Lin⁻: Sca1⁺: CD81⁺
Incubate with
CL316,243 (β3-AR agonist)
Isoproterenol
Norepinephrine
proliferation assay
E
vehicle
CL 316,243 (β3-AR agonist)
Isoproterenol
Norepinephrine
ns
Cell number (x10⁴/well)
(days)
ns
Cell number (x10⁴/well)
vehicle
CL316,243 (β3-AR agonist)
Isoproterenol
Norepinephrine

Supplemental Figure 3. (related to Figure 1): Gating Strategy to Characterize Ki67$^+$ CD81$^+$ APC.

A. A sequential gating strategy to characterize Ki67$^+$ CD81$^+$ cells (Lin$^-$: Sca1$^+$: CD81$^+$: Ki67$^+$) from adipose tissues. After depleting lineage$^+$ (Lin$^+$) cells by MACS® Non-Adipocyte Progenitor Depletion Cocktail and MACS LS columns, the following antibodies were used to quantify Ki67$^+$ CD81$^+$ cells: Sca-1-PB (1:800), CD81-APC (1:50), and Ki67-PE antibody (1:300). Cell population (%) was calculated as frequency of parent.

B. Representative histogram images of FACS analysis of Ki67$^+$ CD81$^+$ cells in the inguinal WAT of C57BL/6J mice at 30°C or 8°C for 4 days.

C. Schematic illustration of the experiments in *Cd81*-lineage reporter mice (*Cd81*-Cre^{ERT2}; Rosa26-mTmG). Tamoxifen was administered intraperitoneally for 5 days prior to cold exposure. Male mice at 10 weeks old were acclimated at 30°C for 2 weeks and exposed to 8°C for 3 days.

D. Schematic illustration of cell proliferation assay. Inguinal WAT-derived primary CD81$^+$ cells were cultured and treated with vehicle, CL316,243, isoproterenol, or norepinephrine.

E. Left: Changes in the number of CD81$^+$ cells in (D) at indicated time points. n = 4. ns, not significant, by two-way repeated-measures ANOVA. Right: The number of CD81$^+$ cells at 4 days after treatment. n = 4. Data are mean ± SEM.; ns, not significant, by one-way ANOVA followed by the Tukey-Kramer's post hoc test.

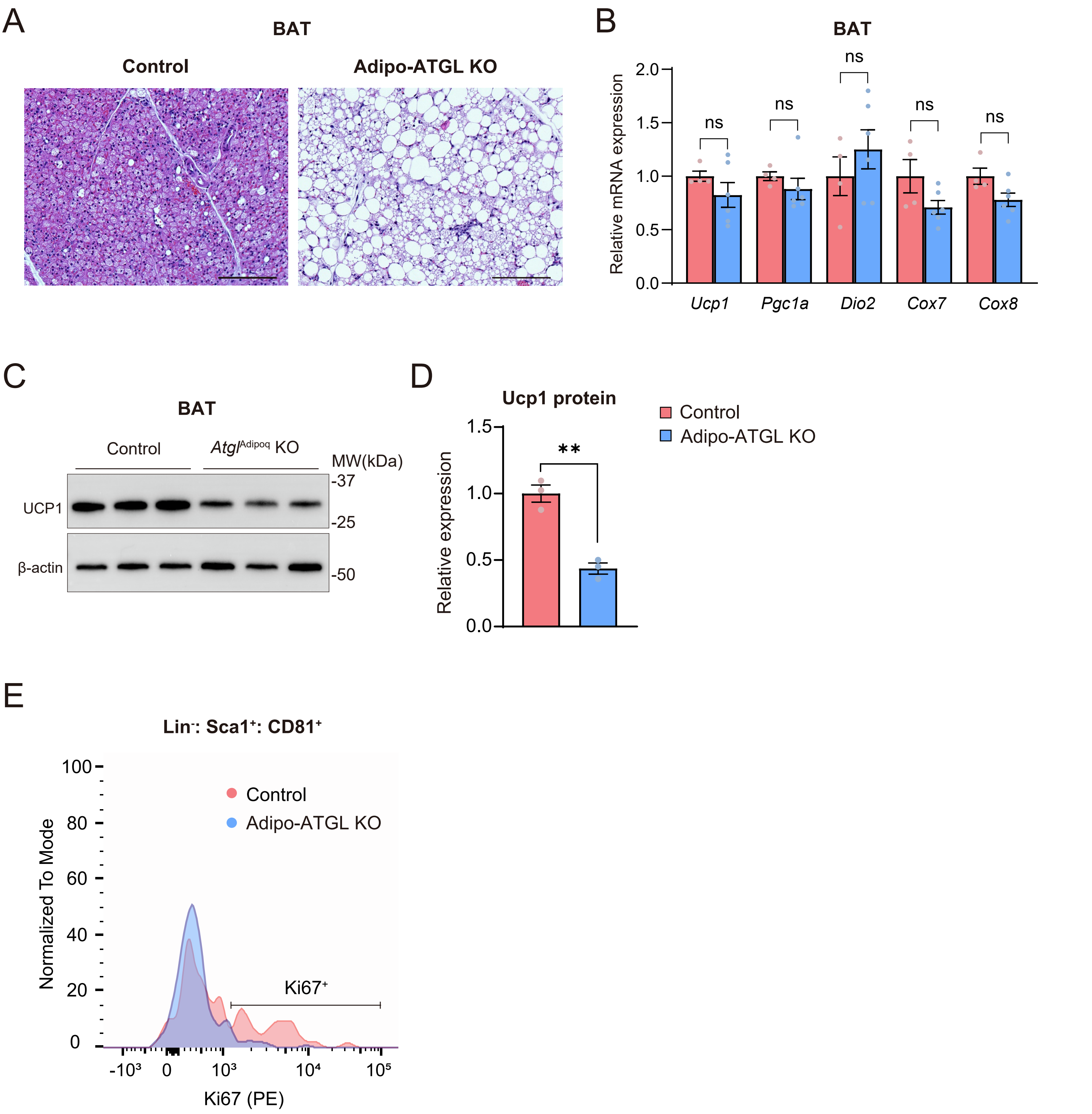

Supplemental Figure 4. (related to Figure 3): Thermogenic Phenotype in the Interscapular BAT of Adipo-ATGL KO mice.

A. Representative H&E staining in the interscapular BAT of mice following cold exposure at 8°C for 3 days. Scale bar, 100 μm.
B. Relative mRNA levels of indicated genes in the interscapular BAT of mice in (A). $n = 6$ for Adipo-ATGL KO mice, $n = 4$ for controls.
C. Immunoblotting of UCP1 in the iBAT of mice in (A). Molecular weight (kDa).
D. Immunoblotting-based quantification of UCP1 protein in (C). $n = 3$.
E. Representative FACS histogram images of Ki67^{+} CD81^{+} cell population in the inguinal WAT of Adipo-ATGL KO mice and control mice following cold exposure at 8°C.
B, D. Data are mean ± SEM.; ** $p < 0.01$, ns, not significant, by two-tailed unpaired Student's *t*-test.

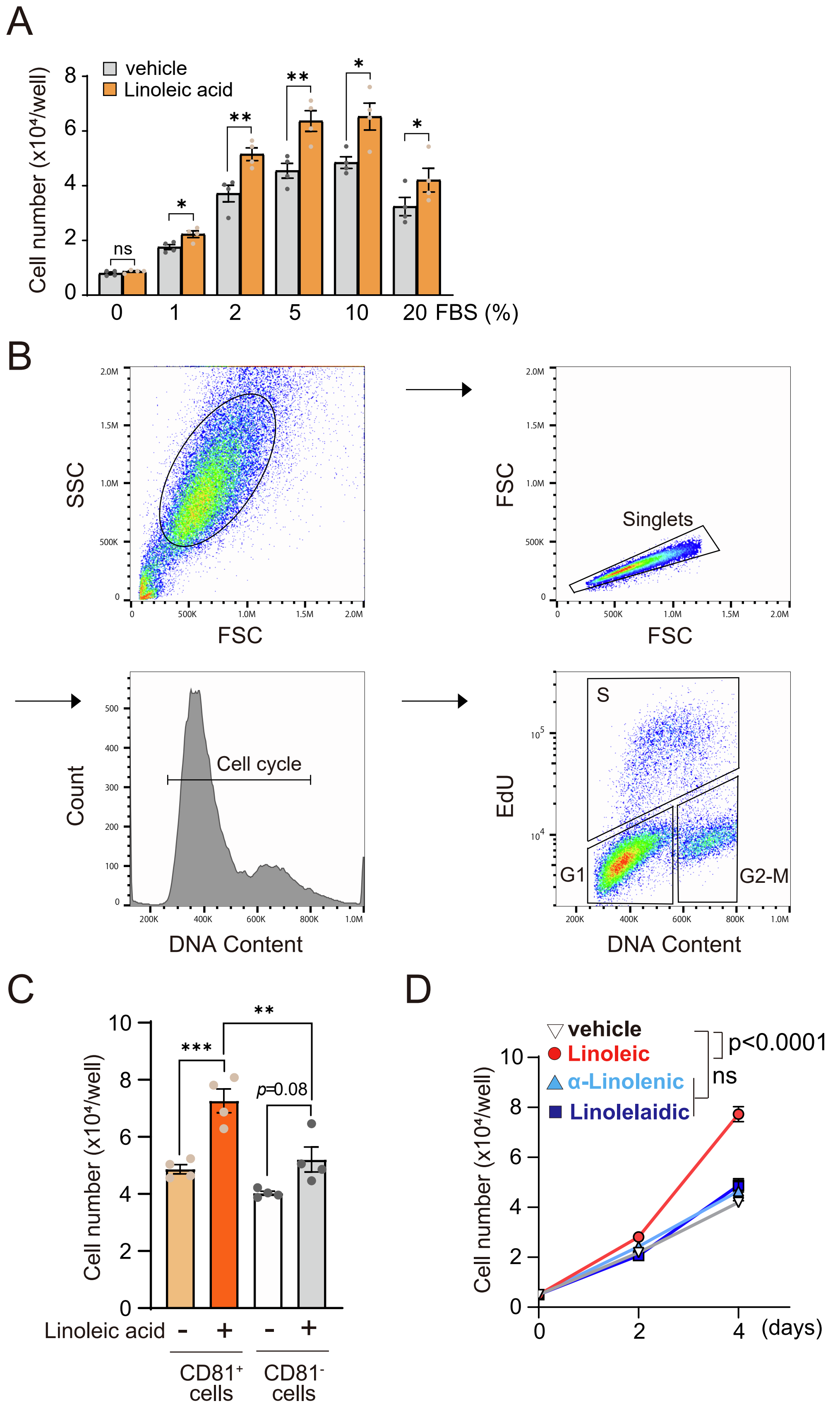

Supplemental Figure 5. (related to Figure 4): Linoleic Acid Treatment Promotes CD81⁺ APC Proliferation.

A. Changes in the number of inguinal WAT-derived CD81⁺ cells cultured in the media containing the indicated concentrations of FBS. Cells were also treated with BSA-conjugated linoleic acid for 4 days. $n = 4$. * $p < 0.05$, ** $p < 0.01$, by two-tailed unpaired Student's *t*-test.

B. A sequential gating strategy for determining cell-cycle phases of CD81⁺ cells. The number of cells in each cell-cycle phase was quantified based on the signals of EdU uptake and DNA content.

C. The number of inguinal WAT-derived CD81⁻ and CD81⁺ cells that were treated with linoleic acid for 4 days. $n = 4$. ** $p < 0.01$, *** $p < 0.001$, by one-way ANOVA followed by the Tukey-Kramer's post hoc test.

D. Changes in the number of inguinal WAT-derived CD81⁺ cells treated with vehicle, linoleic acid, α-linolenic acid, and linolelaidic acid at 5 μM. $n = 4$.; *p*-value was determined by two-way repeated-measures ANOVA.

A, C, D. Data are mean ± SEM. ns, not significant.

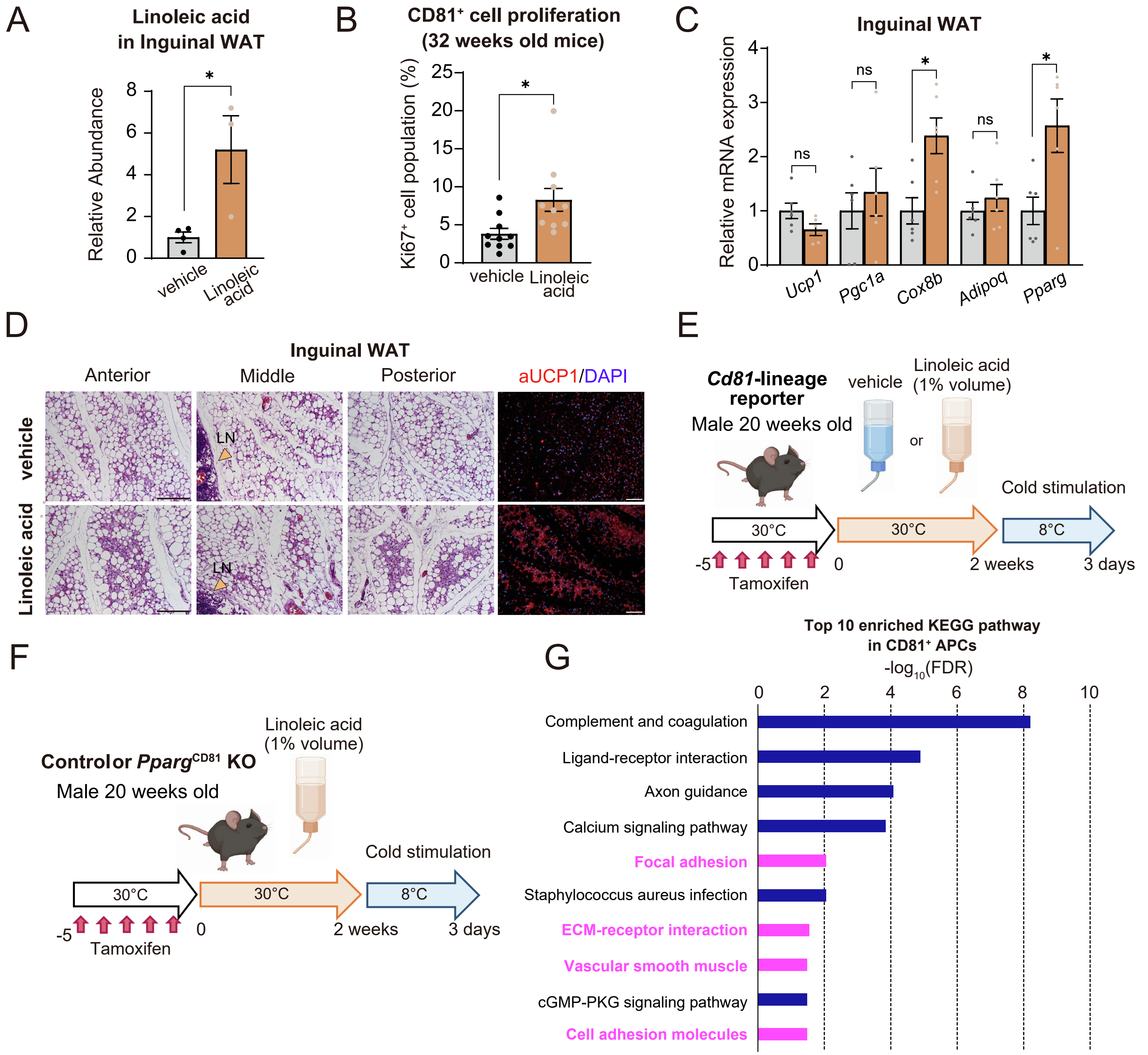

Supplemental Figure 6. (related to Figure 4): Linoleic Acid Supplementation Promotes CD81⁺ APC Proliferation.

A. Relative abundance of linoleic acid in the inguinal WAT of mice following linoleic acid supplementation at 30°C. $n = 4$ for vehicle, $n = 3$ for linoleic acid.

B. FACS-based quantification of Ki67⁺ CD81⁺ cells in the inguinal WAT of mice. Mice at 30 weeks old were orally supplemented with vehicle or linoleic acid (1% volume) at 30°C for 2 weeks. $n = 10$.

C. Relative mRNA levels of indicated genes in the inguinal WAT of mice supplemented with linoleic acid or vehicle for 2 weeks at 30°C. $n = 6$.

D. Representative H&E staining and immunofluorescent staining for UCP1 (Red)/DAPI (Blue) in the inguinal WAT of mice following linoleic acid supplementation and cold exposure. LN: lymph node. Scale bar, 100 µm.

E. Schematic illustration of oral linoleic acid supplementation in *Cd81*-lineage reporter mice. Tamoxifen was administered intraperitoneally for 5 days prior to oral supplementation. Male mice at 20 weeks old were given access to water bottles containing vehicle or linoleic acid (1% volume) for 2 weeks at 30°C, then exposed to 8°C for 3 days.

F. Schematic illustration of oral linoleic acid supplementation in *Pparg*^{CD81} KO mice and littermate controls. After tamoxifen treatment for 5 days, male mice at 20 weeks old were given access to water bottles containing linoleic acid (1% volume) for 2 weeks at 30°C, then exposed to 8°C for 3 days.

G. (related to Figure 5) The pathway enrichment analysis of transcriptomics in inguinal WAT-derived CD81⁺ cells relative to CD81⁻ cells. The upregulated KEGG pathways were shown. FDR, False discovery rate.

A-C. Data are mean ± SEM.; * $p < 0.05$, by two-tailed unpaired Student's t-test. ns, not significant.

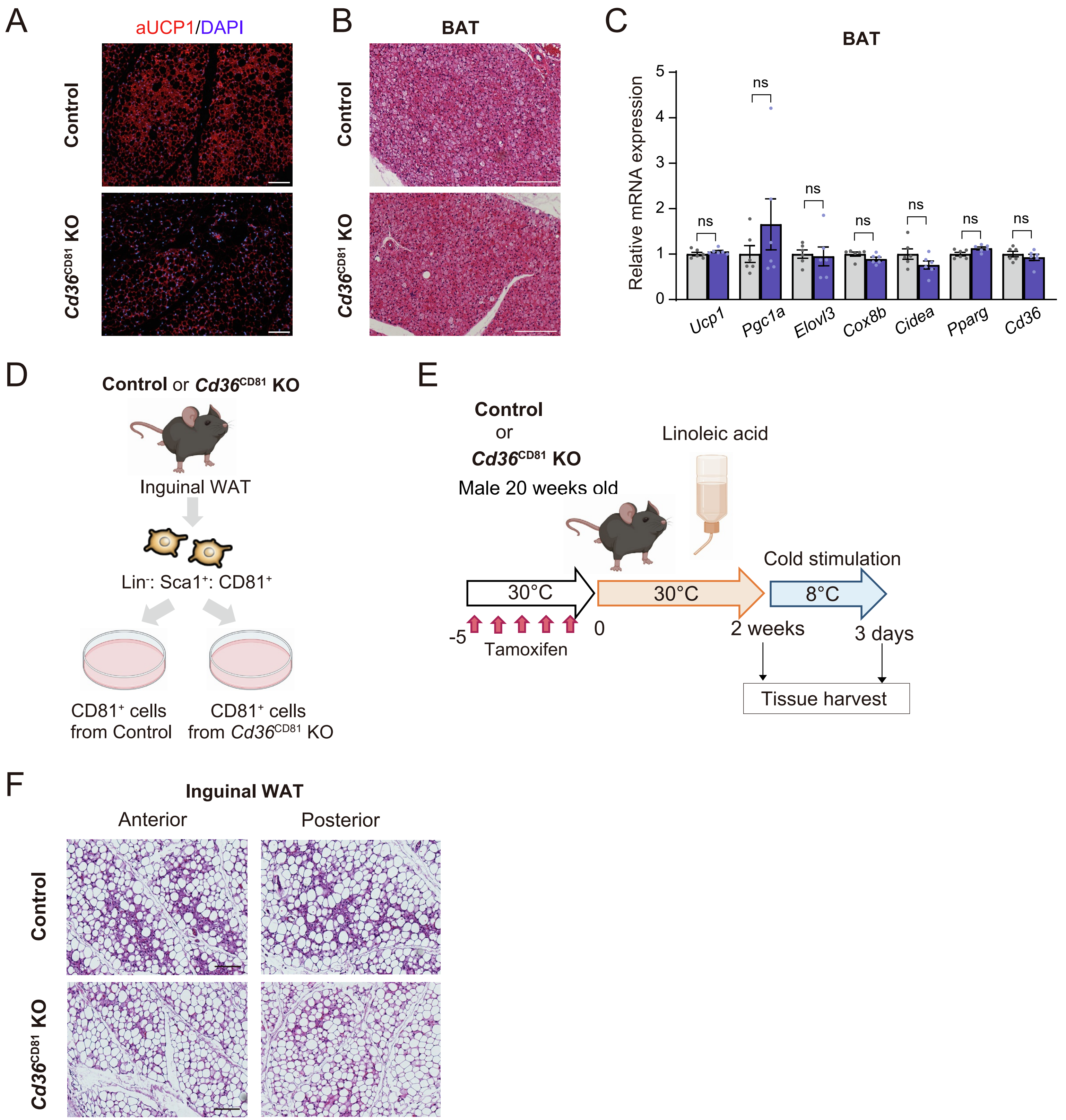

Supplemental Figure 7. (related to Figure 6): Thermogenic Phenotype in the Interscapular BAT of *Cd36*^{CD81} KO mice.

A. Representative immunofluorescent staining for UCP1 (Red)/DAPI (Blue) in the inguinal WAT following cold exposure at 8°C. LN: lymph node. Scale bar, 100 μm.

B. Representative H&E staining of the interscapular BAT at 8°C for 3 days. Scale bar, 100 μm.

C. Relative mRNA levels of indicated genes in the interscapular BAT of mice in (B). $n = 6$. Data are mean ± SEM.; ns, not significant, by two-tailed unpaired Student's *t*-test.

D. Schematic illustration of cell experiments. Primary CD81^{+} cells isolated from the inguinal WAT of *Cd36*^{CD81} KO mice and littermate controls were treated with vehicle or linoleic acid.

E. Schematic illustration of the experiments. *Cd36*^{CD81} KO mice and littermate controls were acclimated to 30°C and received tamoxifen for 5 days prior to linoleic acid supplementation. Mice were given access to water bottles containing vehicle or linoleic acid (1% volume) for 2 weeks at 30°C. A subset of mice was then exposed to 8°C for 3 days.

F. Representative H&E staining (anterior and posterior regions) in the inguinal WAT of mice following cold exposure at 8°C for 3 days. Scale bar, 100 μm.

Supplementary Table 1 (related to Figure 1J): Subject information for ex vivo culture experiments of isolated subcutaneous adipose tissue in human.

Case	BMI	Age	Sex	Ethnicity	Type of adipose tissue	Fat source	Surgery	Institution
1	40.7	57	female	Caucasian	subcutaneous adipose tissue	Abdomen	panniculectomy	BIDMC
2	24.9	46	female	Caucasian	subcutaneous adipose tissue	Perianal	soft tissue removal	BIDMC
3	23.8	60	female	Caucasian	subcutaneous adipose tissue	Abdomen	thighplasty	BIDMC
4	28.1	71	female	Caucasian	subcutaneous adipose tissue	Abdomen	DIEP	BIDMC
5	26.3	50	female	Caucasian	subcutaneous adipose tissue	Abdomen	abdominoplasty	BIDMC
6	28.1	42	female	Caucasian	subcutaneous adipose tissue	Abdomen	abdominoplasty	BIDMC
7	35.4	35	female	Caucasian	subcutaneous adipose tissue	Abdomen	abdominoplasty	BIDMC

BMI; body mass index, DIEP; deep inferior epigastric perforators, BIDMC; beth israel deaconess medical center

Supplementary Table 2 (related to STAR methods): Primer sequences used for quantitative RT-PCR.

Gene	Forward	Reverse
Adipoq	GCACTGGCAAGTTCTACTGCAA	GTAGGTGAAGAGAACGGCCTTGT
Cd36	TGCATTTGCCAATGTCTAGC	CCCTCCAGAATCCAGACAAC
Cd81	TCTACGTGGGCATCTACATTCT	ATCCTTGGCGATCTGGTCTTT
Cidea	ATCACAACTGGCCTGGTTACG	TACTACCCGGTGTCCATTTCT
Cox7a	CAGCGTCATGGTCAGTCTGT	AGAAAACCGTGTGGCAGAGA
Cox8b	GAACCATGAAGCCAACGACT	GCGAAGTTCACAGTGGTTCC
Dio2	CAGTGTGGTGCACGTCTCCAATC	TGAACCAAAGTTGACCACCAG
Elovl3	TCCGCGTTCTCATGTAGGTCT	GGACCTGATGCAACCCTATGA
Fabp4	AAGGTGAAGAGCATCATAACCCT	TCACGCCTTTCATAACACATTCC
Fasn	GGAGGTGGTGATAGCCGGTAT	TGGGTAATCCATAGAGCCCAG
Leptin	GAGACCCCTGTGTCGGTTC	CTGCGTGTGTGAAATGTCATTG
Pgc1a	AGCCGTGACCACTGACAACGAG	GCTGCATGGTTCTGAGTGCTAAG
Pnpla2	GAGTGCAGTGTCCTTCACCA	ATCAGGCAGCCACTCCAAC
Pparg	GCATGGTGCCTTCGCTGA	TGGCATCTCTGTGTCAACCATG
Prdm16	GGCGAGGAAGCTAGCCAAA	GGTCTCCTCCTCGGCACTCT
Ucp1	CACCTTCCCGCTGGACACT	CCCTAGGACACCTTTATACCTAATGG
Tbp	ACCCTTCACCAATGACTCCTATG	TGACTGCAGCAAATCGCTTGG
36B4	TCCAGGCTTTGGGCATCA	CTTTATCAGCTGCACATCACTCAGA
